# Azaborine as a Versatile Weak Donor for Thermally Activated Delayed Fluorescence

**DOI:** 10.1021/acsami.3c05409

**Published:** 2023-05-18

**Authors:** Pagidi Sudhakar, Suman Kuila, Kleitos Stavrou, Andrew Danos, Alexandra M. Z. Slawin, Andrew Monkman, Eli Zysman-Colman

**Affiliations:** † Organic Semiconductor Centre, EaStCHEM School of Chemistry, University of St Andrews, St Andrews, Fife KY16 9ST, United Kingdom; ‡ Department of Physics, Durham University, Durham, DH1 3LE, U.K.

**Keywords:** azaborine donor, thermally activated delayed fluorescence, donor−acceptor, triazine, organic light-emitting diodes

## Abstract

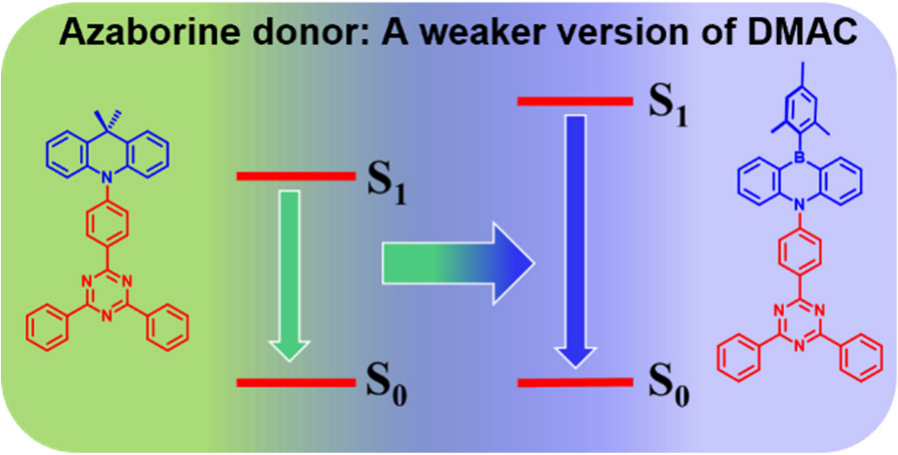

Extensive research has been devoted to the development of thermally activated delayed fluorescence emitters, especially those showing pure-blue emission for use in lighting and full-color display applications. Toward that goal, herein we report a novel weak donor, 1,4-azaborine (AZB), with complementary electronic and structural properties compared to the widely used dimethylacridan (DMAC) or carbazole (Cz) donors. Coupled with a triazine acceptor, **AZB-Ph-TRZ** is the direct structural analogue of the high-performance and well-studied green TADF emitter **DMAC-TRZ** and has Δ*E*_ST_ = 0.39 eV, a photoluminescence quantum yield (Φ_PL_) of 27%, and λ_PL_ = 415 nm in 10 wt % doped mCP films. The shortened analogue **AZB-TRZ** possesses red-shifted emission with a reduced singlet–triplet gap (Δ*E*_ST_ = 0.01 eV) and fast reverse intersystem crossing (*k*_RISC_ of 5 × 10^6^ s^–1^) in mCP. Despite a moderate Φ_PL_ of 34%, OLEDs with **AZB-TRZ** in mCP showed sky-blue emission with CIE_1931_(*x,y*) of (0.22,0.39) and a maximum external quantum efficiency (EQE_max_) of 10.5%. Expanding the chemist’s toolkit for the design of blue donor–acceptor TADF materials will enable yet further advances in the future, as AZB is paired with a wider range of acceptor groups.

## Introduction

Flat panel displays and lighting based on organic light-emitting diodes (OLEDs) show high efficiencies, are ultrathin, lightweight, and flexible, and can exhibit desirable saturated color coordinates including true black.^1−4^ A decade ago, thermally activated delayed fluorescence (TADF) was reintroduced to the organic semiconductor community by Adachi and co-workers, who demonstrated its great potential in harvesting nonemissive triplet excitons and produced OLEDs with efficiencies comparable to those of commercialized versions using organometallic phosphorescent emitters.^5,6^ Indeed, TADF OLEDs can achieve up to 100% internal quantum efficiency (IQE), as the emitter is capable of harnessing all the singlet and triplet excitons and converting these to light via emission from the singlet excited state.

In twisted donor–acceptor (D-A) materials, TADF is activated when there is a sufficiently small energy gap (Δ*E*_ST_) between the lowest-lying singlet (S_1_) and triplet (T_1_) states, which is achieved by spatially separating the electron densities of the HOMO and LUMO of the molecule on the donor and acceptor moieties, respectively. In addition to the conformation adopted, the electron-donating/-accepting strength of the donor/acceptor moieties strongly influences the degree of localization of the frontier molecular orbitals and hence the emission color of the resulting charge-transfer (CT) emission.^7−9^ Blue emitters require a large HOMO–LUMO energy gap, which necessitates the use of both weak donor and acceptor groups.^10,11^ A consequence of the combination of weak donor and acceptor groups is extremely weak excited state conjugation across the D-A molecule, which together with the near-perpendicular D-A geometries further hinders the S_1_ → S_0_ oscillator strength. Simultaneously, low CT character in these excited states can hinder the spin-vibronic coupling mechanism that drives reverse intersystem crossing (RISC).^12,13^ Hence, blue D-A TADF emitters can often suffer from low photoluminescence quantum yields (Φ_PL_) and sometimes display minimal or even no TADF due to an imbalance in donor and acceptor strengths.^10^ As an illustrative example, D-A materials comprising a benzonitrile acceptor and dimethylacridan (DMAC) as the donor display strong (yet green) TADF,^14,15^ while analogues using the weaker donor carbazole (Cz) only exhibit blue room-temperature phosphorescence.^16^ Therefore, for the continued development of blue TADF emitters, a wider selection of appropriate donors and acceptors is critical.

**Figure 1:**
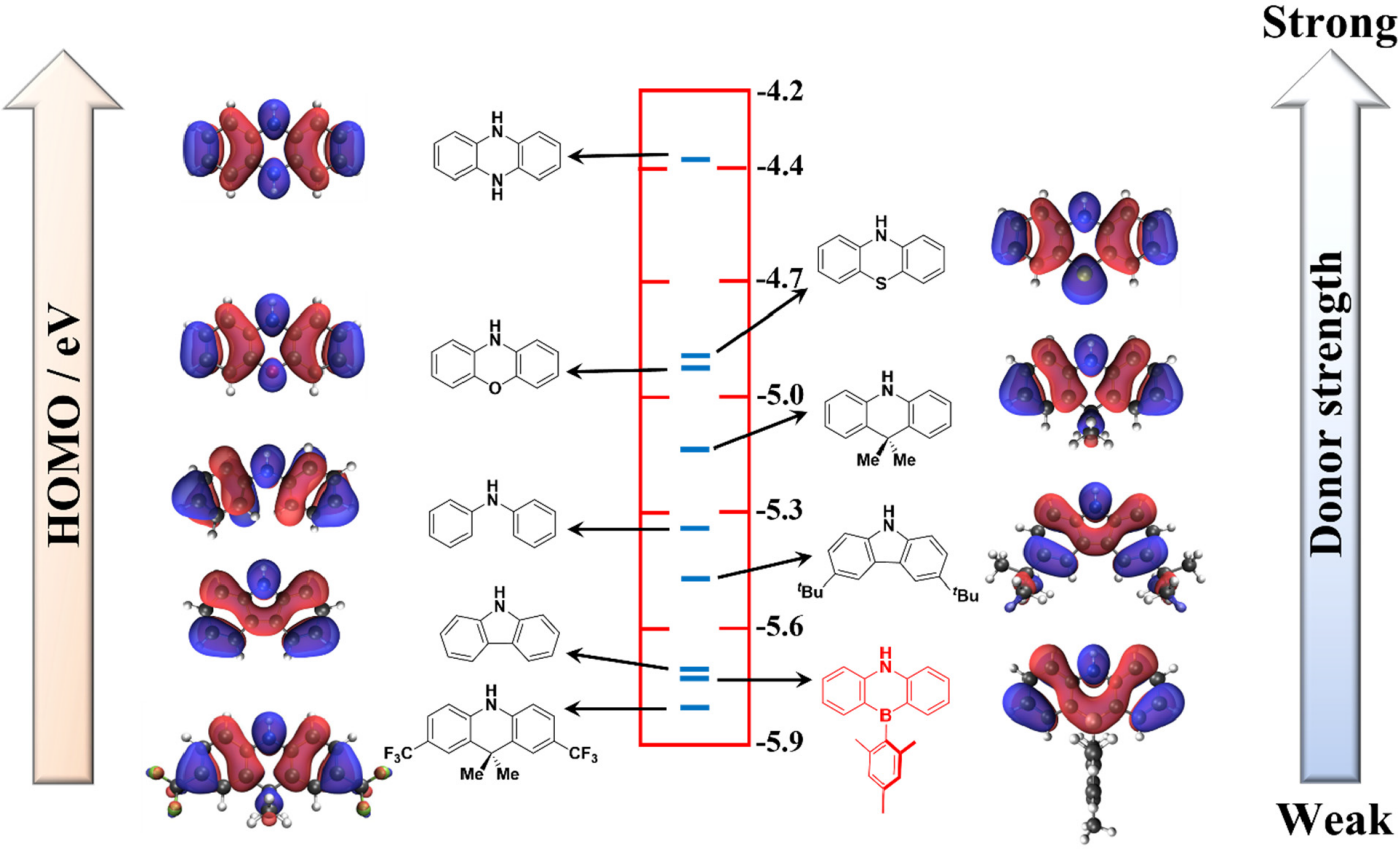
Structures, HOMO electron density distributions, and HOMO energy levels of commonly used N-heterocyclic electron donors (black) and AZB (red), computed at the PBE0/6-31G(d,p) level of theory (isovalue = 0.02).

Some of the most commonly used electron donors in TADF emitter design include Cz (*E*_HOMO_ = −5.73 eV, 3,6-di-*tert*-butylcarbazole, dtBuCz, *E*_HOMO_ = −5.47 eV), diphenylamine (*E*_HOMO_ = −5.34 eV), DMAC (*E*_HOMO_ = −5.13 eV, 9,9-dimethyl-2,7-bis(trifluoromethyl)-9,10-dihydroacridine, *E*_HOMO_ = −5.84 eV), phenoxazine (*E*_HOMO_ = −4.92 eV), phenothiazine (*E*_HOMO_ = −4.89 eV), and 5,10-dihydrophenazine (*E*_HOMO_ = −4.38 eV). Of these, only Cz and DMAC and their derivatives are suitably weak to be used in blue TADF emitters (Figure 1), with Cz alone of these being suitable for deep-blue or UV-emissive D-A TADF emitters.^17−19^ However, the compact size of Cz results in compounds adopting less-twisted conformations, leading to emitters with undesirably large Δ*E*_ST_ and subsequently weak or even inactive TADF. Indeed, the D-A dihedral angles of Cz-containing TADF materials are significantly impacted by their steric environment, for example taking on significantly more planar conformations when attached to compact heterocycles such as pyridine, pyrimidine, or pyrazine, in contrast to more twisted conformations when attached to phenylenes.^20,21^ Cz-containing TADF emitters have also been shown to form persistent dimer states that can be detrimental to emission color, color purity, and Φ_PL_.^22^ In contrast, the larger 6-membered central ring of DMAC results in near-perpendicular conformations that are less sensitive to the steric environment,^14,23^ although it is simultaneously a much stronger electron donor.^24^

It would therefore be appealing to design weak donors akin to Cz but that share the useful steric properties associated with DMAC.^9^ Previously we have reported a CF_3_-substituted DMAC derivative that is a weaker electron donor due to inductively electron-withdrawing trifluoromethyl groups, which yielded a blue-shifted emission compared to the reference DMAC-based material; however, this emitter was electrically unstable in an OLED.^9^ Here, we envision an embedded boron atom within the donor acting as an electron-withdrawing group, which will similarly subdue the electron-donating strength of the corresponding 1,4-azaborine (AZB).

**Figure 2:**
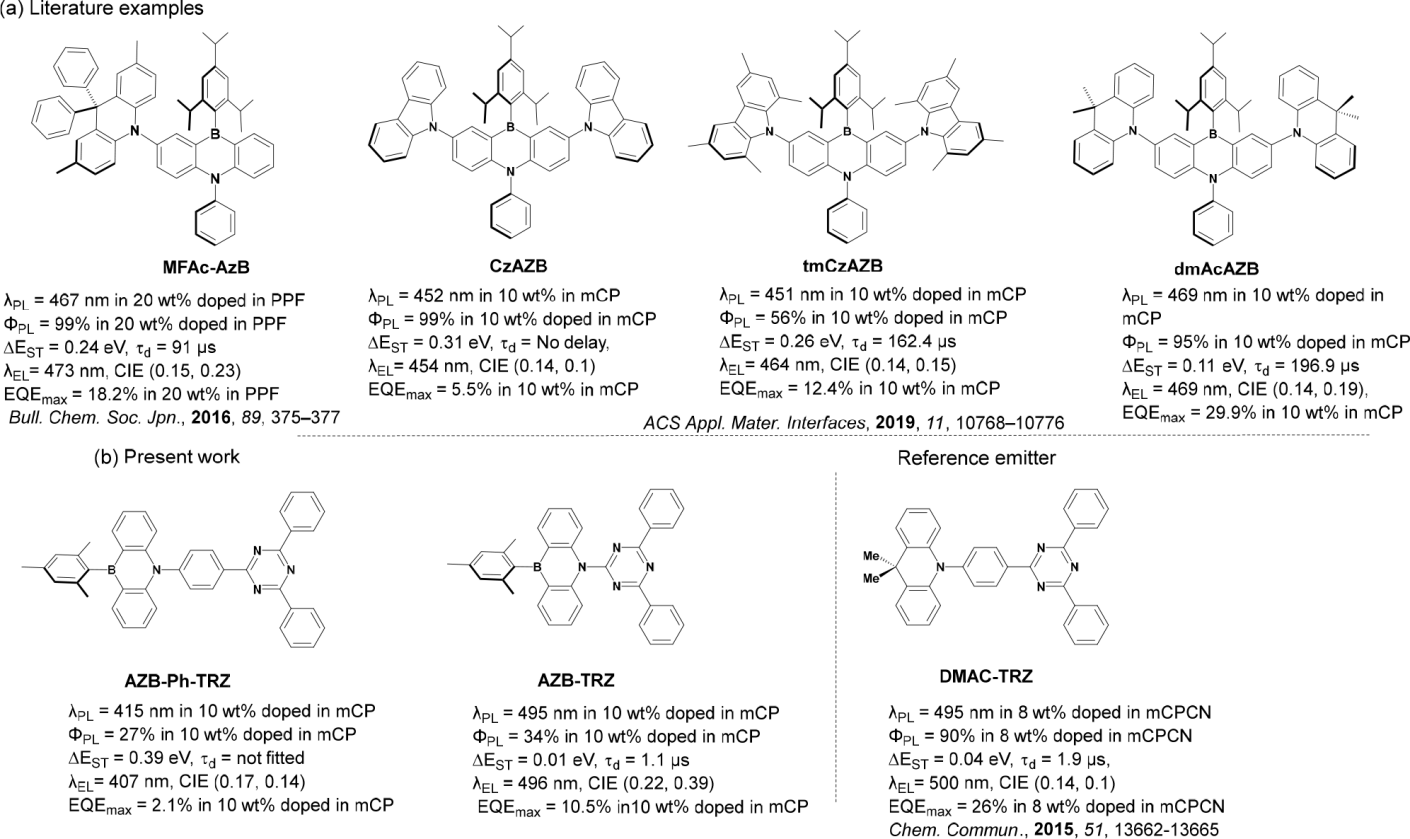
(a) Chemical structures of reported azaborine acceptor-based emitters with their photophysical and OLED data. (b) AZB-based molecules reported in the present study, along with the reference compound, **DMAC-TRZ**.

Park et al.^25^ were the first to report the use of AZB as an acceptor group in a D-A TADF emitter. **MFAc-AzB** exhibits an emission maximum, λ_PL_, of 467 nm and a Φ_PL_ of 99% in a 20 wt % PPF (PPF: 2,8-bis(diphenylphosphine oxide)dibenzofuran) doped film, with an Δ*E*_ST_ of 0.24 eV and a corresponding delayed lifetime (τ_d_) of 91 μs (Figure 2a). The OLED with **MFAc-AzB** showed a maximum external quantum efficiency (EQE_max_) of 18.2% at 473 nm.^25^ Wu et al. nicely demonstrated the impact of donor strength and torsion angle on TADF properties in a series of D-A-D materials with AZB acting as the acceptor.^26^ Although **CzAZB** exhibited a Φ_PL_ of 99%, it did not show TADF, as the Δ*E*_ST_ value of 0.31 eV was too large. This is likely due to the too-planar conformation adopted by the donor moiety, leading to too-strong electronic coupling between the donor and acceptor moieties. However, the use of tetramethylcarbazole (tmCz) and DMAC produced compounds with significantly more twisted conformations, leading to reduced Δ*E*_ST_ values of 0.26 and 0.11 eV for **tmCzAZB** and **dmAcAZB**, respectively, and associated Φ_PL_ values of 56% and 95%, respectively, in 10 wt % doped films in 1,3-bis(*N*-carbazolyl)benzene (mCP). The OLEDs with **tmCzAZB** and **dmAcAZB** showed EQE_max_ values of 12.4% and 29.9%, respectively, at associated λ_EL_ values of 464 and 469 nm (Figure 2a). These and similar studies again highlight the appeal of accessing a weak donor that can enable a blue-shifted emission akin to Cz, but also featuring the useful steric properties associated with DMAC.^27^

In the aforementioned examples AZB was employed as a weak acceptor group, and although azaborine itself has been reported as a fragment in the context of multiresonant TADF emitter design (MR-TADF),^28^ to date its alternate use as an electron donor in conjunction with an appropriately matched electron acceptor has been unexplored.^11^ Here we have hypothesized and subsequently verified both theoretically and experimentally that the electron-deficient boron atom within AZB indeed decreases the donating strength of this donor relative to DMAC, while the size of this donor ensures the desired near-orthogonal conformation in D-A TADF emitters.

Two compounds were targeted (Figure 2b), one with AZB directly coupled with chlorodiphenyltriazine (**AZB**-**TRZ**) and the other coupled to 4-bromophenyldiphenyltriazine (**AZB**-**Ph**-**TRZ**). These materials have been investigated and their properties and devices contrasted to the literature green TADF emitter **DMAC-TRZ** (Figure 2b),^29^ revealing the differences between the AZB and DMAC donors. Note that the analogous **Cz-Ph-TRZ** (Figure 5) is not TADF-active^30,31^ and was not investigated. **AZB-Ph**-**TRZ** and **AZB**-**TRZ** are TADF-active although to significantly different degrees and show deep-blue to sky-blue emission at λ_PL_ values of 415 and 495 nm, respectively, in 10 wt % doped mCP films. OLEDs using the mCP host showed EQE_max_ values of 2.1% and 10.5%, with emission Commission Internationale de l’Éclairage, CIE_1931_(*x,y*), coordinates of (0.17, 0.14) and (0.22, 0.39) for **AZB-Ph**-**TRZ** and **AZB**-**TRZ**, respectively. As it shares desirable electronic and structural features with both Cz and DMAC, we anticipate that use of the AZB donor in combination with other acceptors will become widespread and enable further advances in blue TADF emitter discovery.

## Results and Discussion

### Synthesis and Characterization

**Scheme 1:**
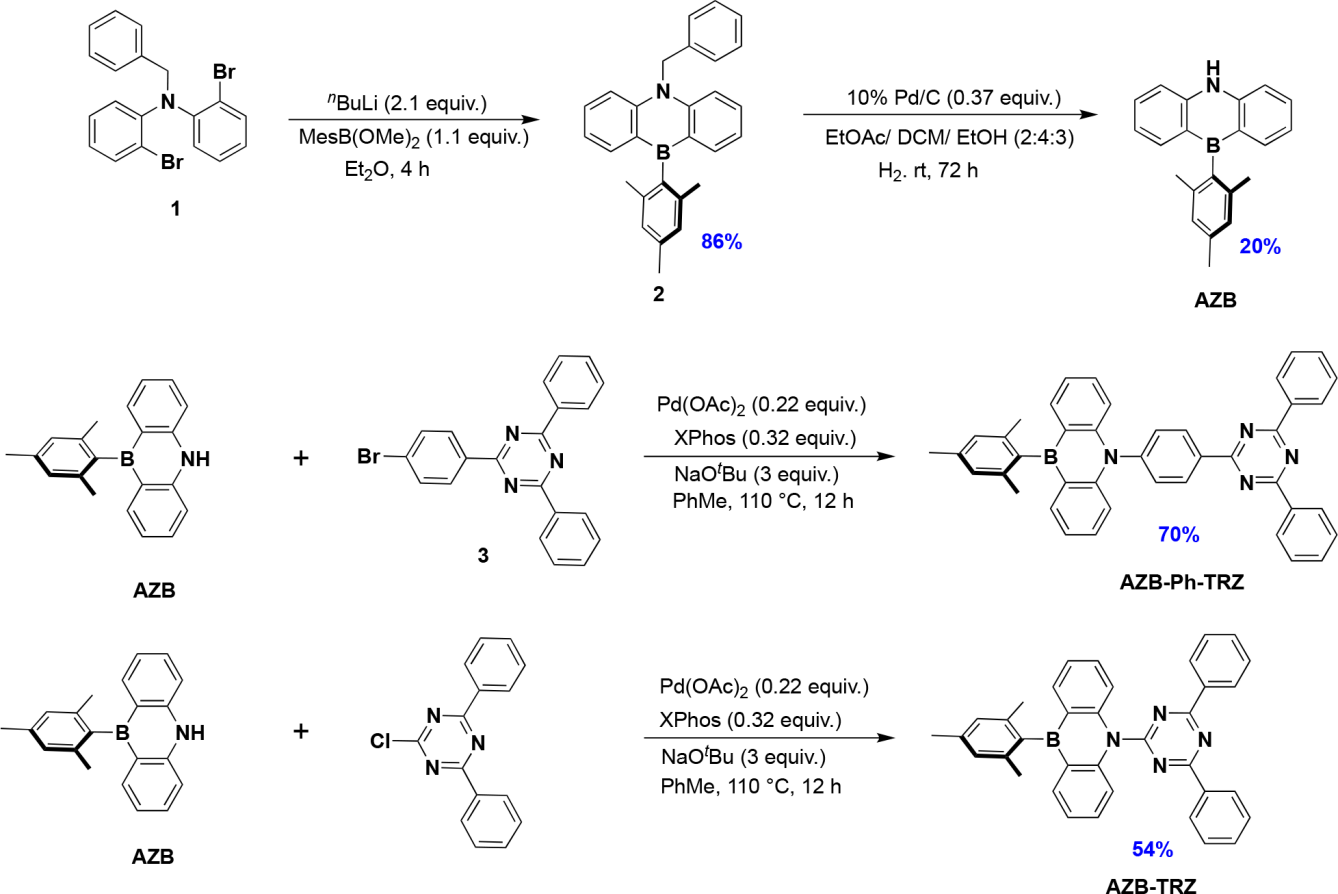
Synthesis of **AZB-Ph-TRZ** and **AZB-TRZ**

The syntheses of **AZB** and the two targeted emitters are shown in Scheme 1. The synthesis of *N*-benzyl-2-bromo-*N*-(2-bromophenyl)aniline^32^ (**1**) and 2-(4-bromophenyl)-4,6-diphenyl-1,3,5-triazine^33^ (**3**) followed literature protocols. The key benzyl-protected precursor, 5-benzyl-10-mesityl-5,10-dihydrodibenzo[*b*,*e*][1,4]azaborinine (**2**), was readily prepared by reacting compound **1** with 2.1 equiv of *n*-butyllithium followed by quenching with dimethyl mesitylboronate. Debenzylation proceeded using Pd/C under a H_2_ atmosphere at room temperature to furnish 10-mesityl-5,10-dihydrodibenzo[*b*,*e*][1,4]azaborinine (AZB) in 20% yield; a significant amount of unreacted starting material was also recovered. The target emitters **AZB-Ph-TRZ** and **AZB-TRZ** were synthesized from AZB and the corresponding triazine derivatives via a Buchwald–Hartwig cross-coupling reaction in moderate-to-good yields. The two compounds were purified first by column chromatography and then by temperature-gradient vacuum sublimation. The identity and purity of the two emitters were determined from a combination of ^1^H and ^13^C NMR spectrometry, high-resolution mass spectrometry (HRMS), elemental analysis (EA), high-performance liquid chromatography (HPLC), and melting point determination (Figures S1–S15). **AZB-Ph-TRZ** and **AZB-TRZ** are thermally stable up to ∼400 and ∼300 °C, respectively, as evidenced by TGA analyses (Figures S11 and S17).

**Figure 3:**
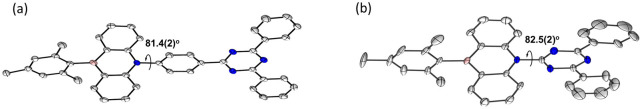
ORTEP molecular structures of (a) **AZB**-**Ph**-**TRZ** and (b) **AZB**-**TRZ** (hydrogen atoms are omitted for clarity; displacement parameters are drawn at the 50% probability level) as determined from X-ray diffraction.

In addition, crystals suitable for X-ray diffraction analysis were obtained directly from the sublimed materials. In the case of **AZB-TRZ**, there are two independent molecules in the asymmetric unit cell. In both compounds, the sum of bond angles around the boron and nitrogen atoms of the AZB are 360°, indicating that each adopts a trigonal-planar geometry, which imparts planarity to the azaborine ring, unlike phenoxazine and phenothiazine. The AZB donor is strongly twisted about the N–C bond, with dihedral angles of 81.4(2)° and 82.5(2)° [87.7(2)°] in **AZB-Ph-TRZ** and **AZB-TRZ**, respectively (Figure 3). This dihedral angle is much larger than the corresponding angle (45.1°) in an equivalent **Cz-Ph-TRZ**(30) analogue and is slightly smaller than the 90° angle in the **DMAC-TRZ**.^34^

### Quantum Mechanical Calculations

We undertook a DFT study to gain an in-depth understanding of the electronic structures of **AZB-Ph-TRZ** and **AZB-TRZ**. The starting geometries of the emitters used for the DFT calculations (PBE0/6-31G(d,p) level of theory in the gas phase) were taken from the X-ray structures. The ground-state optimized geometries of **AZB-Ph-TRZ** and **AZB-TRZ** have a torsion angle of ∼90° between the AZB donor and triazine, while the same torsion angles are slightly smaller in the single-crystal structures, which is attributed to packing forces. The other geometric parameters (bond lengths and angles) of the optimized structures closely match those found in the crystal structures (Figures S18 and S19 and Tables S1 and S2).

**Figure 4:**
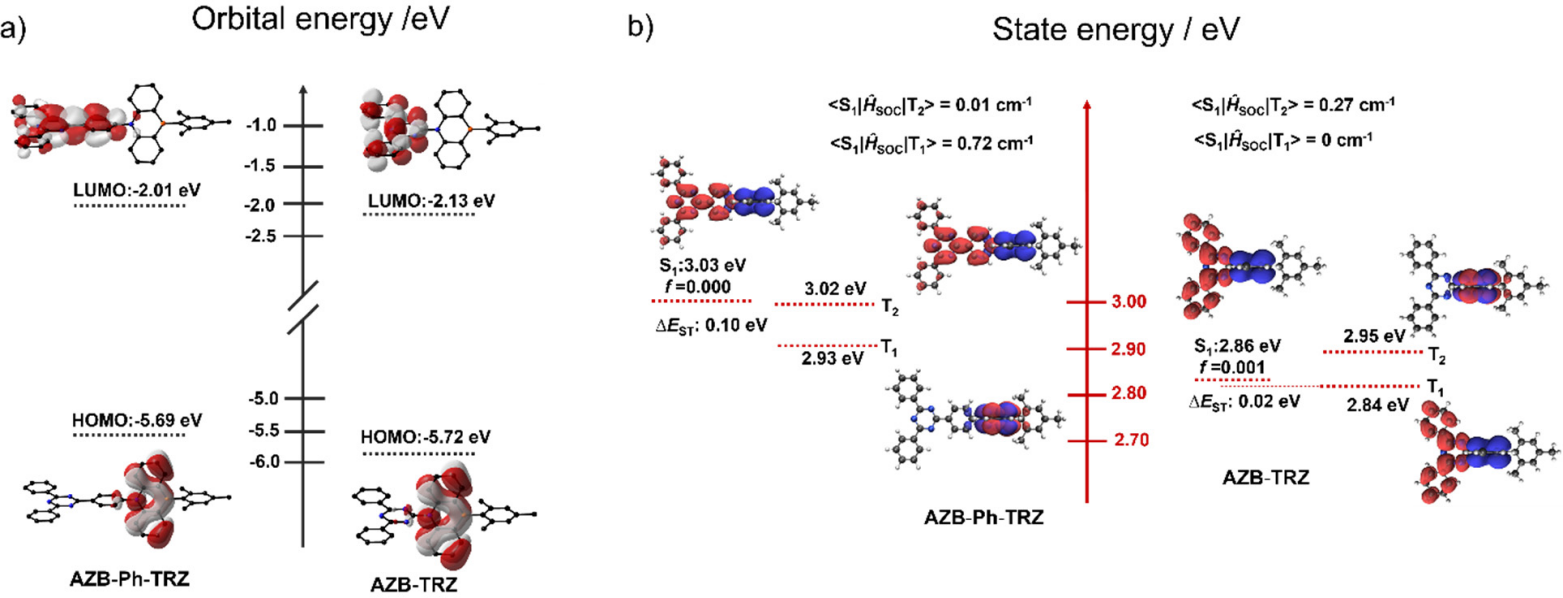
Theoretical modeling of (a) the energies and electron density distributions of the HOMO/LUMO (isovalue = 0.02) and (b) NTOs (particle and hole are represented by red and blue colors, respectively) and their associated vertical excitation energies of S_1_, T_1_, and T_2_ states of **AZB-Ph-TRZ** and **AZB-TRZ** computed based on the ground-state optimized geometries.

Excited-state properties were calculated using time-dependent density functional theory (TD-DFT) within the Tamm–Dancoff approximation (TDA-DFT) based on the optimized ground-state geometries.^35^ The molecular orbitals and their associated energy levels as well as the energies of the lowest-lying excited states are shown in Figure 4. As expected from the perpendicular conformation, the HOMO of each of the two emitters is confined to the AZB moiety while the LUMO is localized on the triazine. In **AZB-Ph-TRZ** the LUMO is also extended onto the bridging phenylene, which forms part of the nonidentical acceptor electronic system. The HOMO and LUMO energies of **AZB-Ph-TRZ** are both destabilized compared to those of **AZB-TRZ**, and considerably more so for the LUMO, corresponding to the distinct diphenyltriazine or triphenyltriazine acceptor units.

Following from recent reports of unexpected multiple conformers in similar DMAC-based emitters,^36,37^ the potential energy surfaces (PESs) for the two compounds were analyzed by performing a relaxed dihedral angle scan between the AZB donor and TRZ acceptor. The lowest energy conformer of each compound has a D-A dihedral angle of 90° (Figures S20 and S21). **AZB-TRZ** has another local minimum energy conformer (with bent donor, dihedral angle ∼190° on the PES). The energy barrier between these two conformers is only 0.088 eV (2.0 kcal mol^–1^), and so rapid interconversion is expected at room temperature. For the bent conformer, the HOMO is mostly located on the mesityl ring attached to the boron with minor contribution from the azaborine ring, while the LUMO is delocalized across both the triazine and azaborine groups (Figure S21).

**Figure 5:**
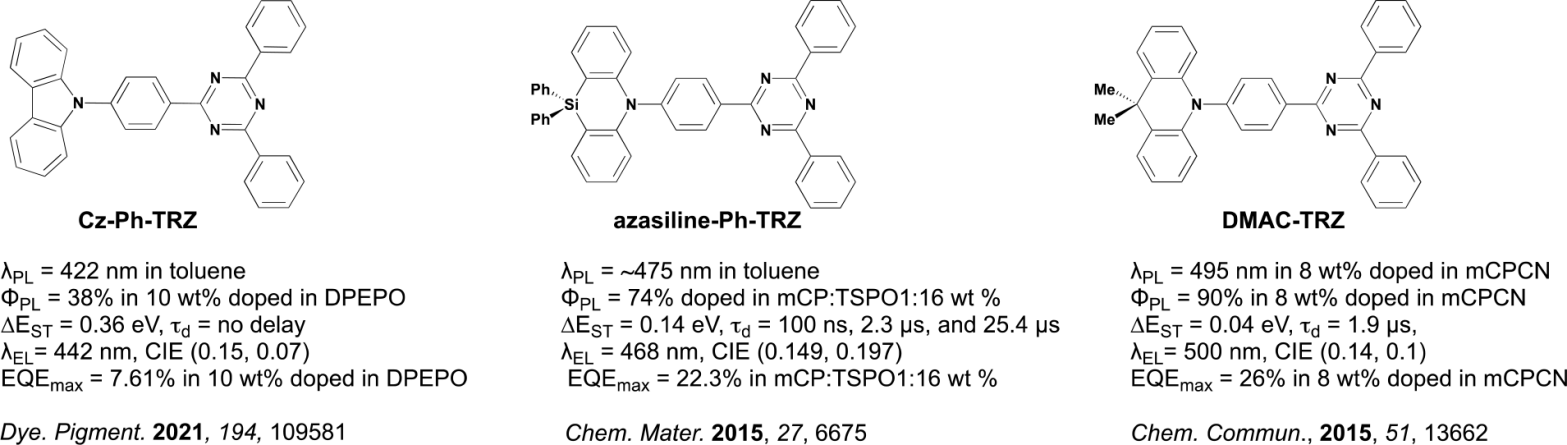
Chemical structures of selected literature donor-TRZ compounds.

The same level of theory was used to additionally compare the HOMO energies of **Cz-Ph-TRZ** (−5.69 eV), **DMAC-TRZ** (−5.13 eV), and **azasiline-Ph-TRZ** (−5.39 eV), each with structures analogous to that of **AZB-Ph-TRZ** (Figure 5). The first of these exactly matches the HOMO level of **AZB-Ph-TRZ** (−5.69 eV), while the HOMO levels of the last two compounds are significantly shallower in energy, evidencing that the AZB donor has indeed a deeper HOMO level (and weaker donating strength) than those of the other six-membered-central-ring DMAC and azasiline counterparts. The HOMO–LUMO energy gap (Δ*E*_HL_) values of 3.68 and 3.59 eV for **AZB-Ph-TRZ** and **AZB-TRZ**, respectively, are consequently larger in comparison to the 2.91 and 3.17 eV for **DMAC-TRZ** and **azasiline-Ph-TRZ** (also known as **DTPDDA**(38) in the literature), respectively, and are most like the 3.82 eV for **Cz-Ph-TRZ**.

The calculated vertical excited singlet (S_1_) and triplet (T_1_) energies of **AZB-Ph-TRZ** are 3.03 and 2.93 eV, respectively, which are stabilized to 2.86 and 2.84 eV for **AZB-TRZ**. These CT states are also stabilized when stronger donors are used, as is the case with **azasiline-Ph-TRZ** (S_1_ 2.80 eV) and **DMAC-TRZ** (S_1_ 2.56 eV). The corresponding calculated Δ*E*_ST_ values are 0.10 eV for **AZB-Ph-TRZ** and 0.02 eV for **AZB-TRZ**, indicating that these compounds should exhibit TADF. These Δ*E*_ST_ values are similar to those calculated for **azasiline-Ph-TRZ** and **DMAC-TRZ**, which is not surprising given the similar perpendicular conformation adopted (both with Δ*E*_ST_ of 0.01 eV). The Δ*E*_ST_ of **Cz-Ph-TRZ** is much larger at 0.38 eV, the result of its less twisted structure. The estimated oscillator strengths (*f*) for the S_0_ → S_1_ transitions (without accounting for vibronic coupling) are 0 and 0.0013 for **AZB-Ph-TRZ** and **AZB-TRZ**, respectively, reflecting the very weak electronic coupling between donor and acceptor groups, due to the near-orthogonal conformation in the compounds.

To gain greater insight into the nature of the excited states, natural transition orbitals (NTOs) were analyzed at the ground-state optimized geometries (Figure 4). Both the T_1_ hole and particle are localized on the donor for **AZB-Ph-TRZ**, indicating localized excitonic (LE) character for this state. For the T_2_ state, the hole and particle are situated on the AZB and triazine-phenylene bridge, respectively, clearly indicating CT character. For **AZB-TRZ** the orbital nature of T_1_ and T_2_ is reversed, and both molecules possess S_1_ states with the expected CT character. Spin–orbit coupling (SOC) values were also calculated based on the optimized T_1_ geometries. Due to the difference in orbital type between the triplet states and S_1_, the SOC is considerably larger for T_1_ (with ⟨S_1_|*Ĥ*_SOC_|T_1_⟩ = 0.72 cm^–1^) than for T_2_ (with ⟨S_1_|*Ĥ*_SOC_|T_2_⟩ = 0.01 cm^–1^) in **AZB-Ph-TRZ**, reflecting El-Sayed’s rule for direct RISC between T_1_ and S_1_.^39^**AZB-TRZ** behaves in a complementary manner, with ⟨S_1_|*Ĥ*_SOC_|T_2_⟩ = 0.27 cm^–1^, which is larger compared to ⟨S_1_|*Ĥ*_SOC_|T_1_⟩ = 0.0 cm^–1^. For **AZB-Ph-TRZ**, T_1_ and T_2_ lie just below the S_1_ state, with the energy differences between S_1_-T_1_, S_1_-T_2_, and T_2_-T_1_ being 0.10, 0.01, and 0.09 eV, respectively. These energy gaps are sufficiently small to support efficient RISC through the spin-vibronic coupling between T_2_ and T_1_ states to the S_1_ state.^40^ In the case of **AZB-TRZ**, T_2_ is slightly higher in energy than S_1_ and the energy differences between S_1_-T_1_, S_1_-T_2_, and T_2_-T_1_ are 0.02, −0.09, and 0.11 eV, respectively. A similar spin-vibronic mechanism is therefore also expected in this compound to support efficient RISC.

### Electrochemistry

**Figure 6:**
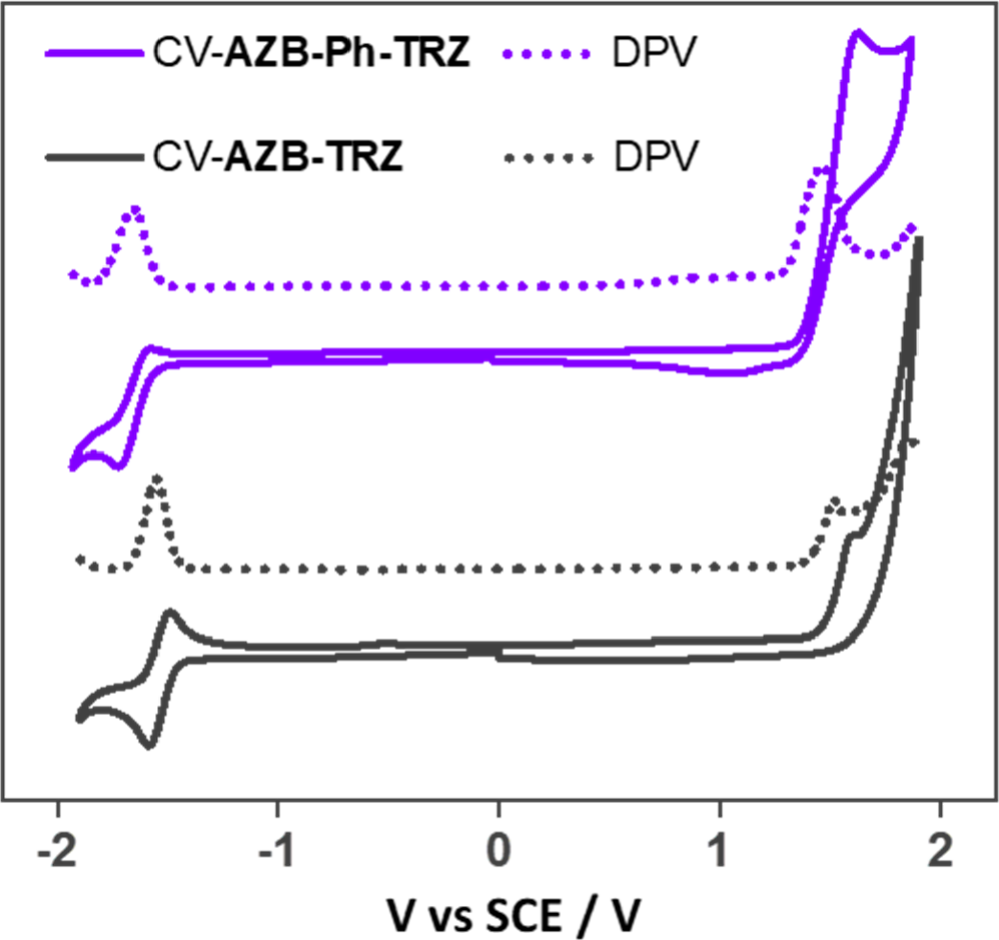
Cyclic voltammograms (CV) and differential pulse voltammograms (DPV) of **AZB-Ph-TRZ** and **AZB-TRZ** in N_2_-saturated DCM solution with 0.1 M [^*n*^Bu_4_N]PF_6_ as the supporting electrolyte and Fc/Fc^+^ as the internal reference (0.46 V for DCM vs SCE).^42^

Cyclic and differential pulse voltammetry (CV and DPV) in dichloromethane were conducted to ascertain experimentally the HOMO and LUMO levels (Figure 6). At a scan rate of 100 mV s^–1^, both compounds showed reversible reduction waves and irreversible oxidation waves. The oxidation potentials obtained from the maxima of the DPVs are 1.46 and 1.51 V, while the reduction potentials are at −1.65 and −1.55 V for **AZB-Ph-TRZ** and **AZB-TRZ**, respectively. The HOMO levels were thus estimated to be −5.79 and −5.85 eV and the LUMO levels were 2.69 and 2.79 eV, respectively. The estimated redox gaps of 3.10 and 3.06 eV reflect the calculated trends in the HOMO–LUMO gaps of 3.68 (**AZB-Ph-TRZ**) and 3.59 eV (**AZB-TRZ**). In comparison, the reported oxidation potentials of **Cz-Ph-TRZ** (also known as **Cz-TRZ**(27) in the literature), **DMAC-TRZ**,^41^ and **azasiline-Ph-TRZ** (also known as **DTPDDA**(38) in the literature, Figure 5) are 1.33, 0.98, and 1.14 V, respectively, which are all significantly cathodically shifted with respect to the two AZB-containing compounds and clearly evidence the weak nature of the azaborine donor and its poor conjugation with the rest of the compound (Table 1).

**Table 1:** Electrochemical Data of **AZB**-**Ph**-**TRZ** and **AZB**-**TRZ** Compounds

emitter	*E*^ox^/V^a^	*E*^red^/V^a^	HOMO/eV^b^	LUMO/eV^b^	Δ*E*_HL_/eV^c^
**AZB**-**Ph**-**TRZ**	1.45	–1.65	–5.79	–2.69	3.10
**AZB**-**TRZ**	1.51	–1.55	–5.85	–2.79	3.06
**Cz-Ph-TRZ**(27)^,^^d^	1.33	–1.55	–5.67	–2.80	2.87
**DMAC-TRZ**(41)	0.98	–1.47	–5.30	–2.78	2.52
**azasiline-Ph-TRZ**(38)^,^^e^	1.14	–1.63	–5.58	–2.72	2.86

a Obtained from DPV peaks and referenced with respect to SCE (Fc/Fc^+^ = 0.46 V for DCM).

b *E*_HOMO/LUMO_ = −(E^ox/red^ (vs Fc/Fc^+^) + 4.8) eV.

c Δ*E*_HL_ = |*E*_HOMO_ – *E*_LUMO_|.^43^

d Obtained from the CV peaks maxima and referenced with respect to SCE (Fc/Fc^+^ = 0.46 V for DCM, used for the oxidation scan; 0.45 V in DMF, used for the reduction scan). **Cz-Ph-TRZ** is known as **Cz-TRZ** in the literature

e Onset potentials referenced with respect to SCE (Fc/Fc^+^ = 0.36 V for CHCl_3_).^44^**azasiline-Ph-TRZ** is known as **DTPDDA** in the literature.

### Photophysical Properties

**Figure 7:**
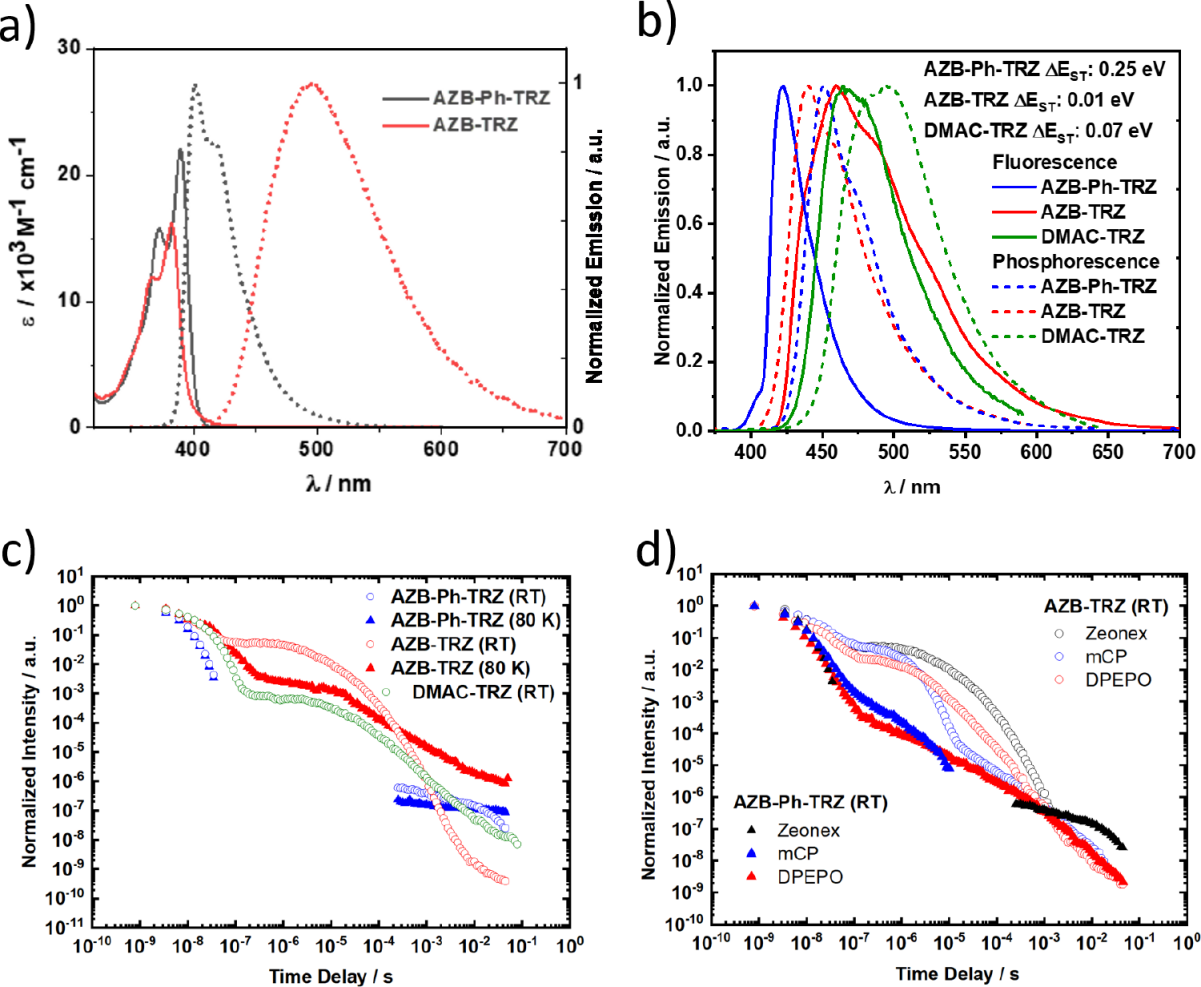
(a) UV–vis absorption and photoluminescence at room temperature in toluene. λ_exc_ = 340 nm. (b) Steady-state photoluminescence (ambient) and phosphorescence (80 K, 80 ms delay) in 1 wt % Zeonex films. (c) Temperature-dependent time-resolved emission decays in Zeonex films (1 wt %). (d) Emission decays of **AZB-Ph-TRZ** and **AZB-TRZ** in Zeonex (1 wt %) and mCP and DPEPO hosts (10 wt %).

Progressing to the optical properties of **AZB-Ph-TRZ** and **AZB-TRZ**, the absorption and emission spectra in toluene are shown in Figure 7a. Absorption maxima at 389 nm (ε = 22 × 10^3^ M^–1^ cm^–1^) and 382 nm (ε = 16 × 10^3^ M^–1^ cm^–1^) for **AZB-Ph-TRZ** and **AZB-TRZ**, respectively, are assigned to π → π* transitions on the AZB fragment, as they match the absorption spectrum of the AZB donor itself (Figure S22). CT absorption bands are practically absent due to the strong electronic decoupling of donor and acceptor moieties in the ground state, although there is a noticeable low-energy foot in the absorption spectrum of **AZB**-**TRZ** (Figure 7a), which we suggest arises from the greater electronic coupling in this compound due to the reduced distance between the donor and acceptor moieties, a picture that aligns with the DFT study.^45,46^

In toluene **AZB-Ph-TRZ** emits at λ_PL_ = 400 nm along with a weak shoulder band around 417 nm, indicating emission from an LE state that matches that of the donor (Figure S22). **AZB-TRZ** instead exhibits a broad and red-shifted CT emission at λ_PL_ = 494 nm. In toluene, **Cz-Ph-TRZ, DMAC-TRZ**, and **azasiline-Ph-TRZ** emit at 422, 498, and ∼460 nm, respectively, all of which are red-shifted compared to the λ_PL_ of **AZB-Ph-TRZ**.^27,34,38^ TD-DFT calculations on the S_1_-optimized geometries predict λ_PL_ of 472 nm (*f* = 0.000) and 573 nm (*f* = 0.001) for **AZB-Ph-TRZ** and **AZB-TRZ** in the gas phase, respectively, which align with these experimental results. The emission spectra of **AZB-Ph-TRZ** and **AZB-TRZ** both show typical bathochromic shifts with increasing solvent polarity (positive solvatochromism), indicating that the S_1_ state is CT in nature in sufficiently polar media (Figure S23). Comparing the emission onset wavelengths of the structurally analogous series in more polar THF solvent (required to elicit only CT emission), we identify that **Cz-Ph-TRZ** has the shortest wavelength (highest energy) onset at 400 nm, followed by **AZB-Ph-TRZ** (425 nm) and then **azasilane-Ph-TRZ** (450 nm).^27,38^ This comparison of emission onsets largely reveals the order of donor strengths, although the distinct donor steric environment and consequently lower CT character of **Cz-Ph-TRZ** makes it less sensitive to host polarity and blue-shifts its emission more than might be expected based on the strength of the donor from calculations alone (Figure 1). Similar comparisons of reported emission onsets between **azasilane-Ph-TRZ** and **DMAC-TRZ** in DCM place the donor strength of DMAC as yet stronger than azasilane.

The Φ_PL_ values of **AZB-Ph-TRZ** and **AZB-TRZ** in degassed toluene solutions are 48% and 13%, respectively, which decreased to 40% and 6% upon exposure to oxygen. The time-resolved emission decay for **AZB-Ph-TRZ** consists of only a prompt component (∼4 ns lifetime), consistent with its LE emission character and oxygen-insensitive Φ_PL_ in this solvent. **AZB-TRZ** in toluene instead decays with biexponential kinetics, with a prompt lifetime, τ_p_, of 28 ns (60%) and a very short delayed lifetime, τ_d_, of 117 ns (40%). Upon exposure to oxygen, the τ_p_ shortened to 13 ns (62%) while the τ_d_ decreased to 52 ns (38%) (Figure S24). Because the delayed lifetime for **AZB-TRZ** is unusually short and is accompanied by a very low Φ_PL_, this implies significant nonradiative decay in solution as τ_d_ and Φ_PL_ increase significantly in rigid hosts. Measurements in rigid hosts already reveal very small Δ*E*_ST_ values, and so TADF should remain comparably efficient. We thus hypothesize that one possible source of the additional nonradiative decay is the conformational interconversion between the bent and the orthogonal conformers in the excited states of **AZB-TRZ**.

Transitioning to solid hosts, the PL spectra (Figure 7b) and decays (Figures 7c,d) of **AZB-Ph-TRZ** and **AZB-TRZ** were investigated in Zeonex and two high triplet energy OLED-compatible hosts with contrasting polarity: mCP and DPEPO. In 1 wt % doped Zeonex films, **AZB-Ph-TRZ** at 300 K shows very long-lived and weak delayed fluorescence (DF), having two main components with the same spectrum across all of the DF lifetime in addition to the short-lived prompt fluorescence (PF, individual spectra shown in Figure S26). The emission is itself very narrow, indicative of ^1^LE excitons as was observed in toluene. In comparison, **AZB-TRZ** shows much stronger DF with considerably shorter DF lifetimes, along with a longer-lived PF (individual spectra shown in Figure S27 and fitted exponential lifetimes and rate constants in Table S3). The DF for **AZB-TRZ** in Zeonex is considerably longer than in toluene solution though (τ_d_ of 12 μs versus 117 ns), which we attribute to the host matrix preventing interconversion between the bent quenching conformer that otherwise accelerates nonradiative triplet decay.

The steady-state PL spectrum for **AZB-TRZ** is broad but slightly structured, while being Gaussian-shaped in the time-resolved spectra, which we interpret as a mixture of molecules in the film contributing to the steady-state emission with slightly different D-A dihedral angles: some with predominantly ^1^CT DF alongside others emitting with ^1^LE character PF. Slight differences in emission color and lifetimes between different molecules also explains the various red shifts/blue shifts of the overall emission at different delay times (Figure S26).^47,48^ The mixture of LE character emission in both materials reflects the weakness of the AZB donor as well as the nonpolar Zeonex host being unable to strongly stabilize CT states. To account for this mixture, experimental singlet energies are taken from the onsets of the relaxed PF emission spectra (Figures S26 and S27), allowing us to avoid the LE emission component in determining these values (Table S3).

As seen in Zeonex, the TADF behavior of both **AZB-Ph-TRZ** and **AZB-TRZ** varies considerably when dispersed in small-molecule hosts mCP and DPEPO. The emission decays of each emitter:host combination is strikingly different, both when comparing the two emitters in the same host and when comparing a single emitter in each of the three different environments. For both materials, the DF intensity is lower and the DF emission is longer-lived in DPEPO than in mCP. There is also a notable long-lived secondary DF component for **AZB-TRZ** in mCP, distinct from the initial rapidly decaying DF. We have recently demonstrated how the flexibility of some D-A bridging bonds can lead to large conformational distributions of molecular geometries in solid films, with subsets of molecules possessing different TADF properties (especially decay times). Similar to the different contributions to the steady-state emission in Zeonex, we suggest that this secondary DF component arises from a subset of **AZB-TRZ** molecules in an unfavorable conformation in the film, with corresponding long-lived emission.^47^

To reveal the nature of the triplet states in these two derivatives, time-dependent emission measurements were performed at 80 K to measure the phosphorescence emission (PH) and determine the energy of the T_1_ state. We observed a substantial red shift in the delayed emission spectra of **AZB-Ph-TRZ** at long delay times (∼80 ms) in 1 wt % doped Zeonex films, which we interpret as the emergence of the PH spectrum (Figure S26). The microsecond-regime delayed emission is fully suppressed at this low temperature, consistent with a TADF mechanism. Significantly reduced delayed emission intensity was also observed for **AZB-TRZ**, along with PH emission at very long delay times at 80 K (Figure S27). The PH spectrum of **AZB-TRZ** is only subtly different from the emission of the PF or DF, but the emergence of the differently structured emission allows us to attribute it as belonging to an ^3^LE state nonetheless. The **AZB-TRZ** PH can also have a higher energy onset than the steady-state PL (Figure 7b), a consequence of host polarity or polarizability red-shifting the CT-character singlet states while the LE triplet states are unimpacted.^48−50^

Consistent with these decay kinetics, the experimental Δ*E*_ST_ of **AZB-TRZ** in 1 wt % doped Zeonex films (0.01 eV, from the onsets of relaxed PF emission at RT and 80 K PH, corresponding to a ^1^CT-^3^LE gap), is substantially smaller than that of **AZB-Ph-TRZ** (0.25 eV). These Δ*E*_ST_ values qualitatively reproduce the ordering of the DFT-calculated Δ*E*_ST_ gaps, although they are substantially larger for **AZB-Ph-TRZ**. The observed *k*_RISC_ ((5.7 ± 0.2) × 10^6^ s^–1^, from kinetic decay fitting^51^) for **AZB-TRZ** indicates its potential as an emitter in OLEDs (Table S3). Similar trends in Δ*E*_ST_ were found in mCP and DPEPO films, having gaps ≥0.15 eV for **AZB-Ph-TRZ** and ≤0.02 eV for **AZB-TRZ** in both hosts (Table S3). This results in a similarly large *k*_RISC_ of 4.9 ± 0.3 × 10^6^ s^–1^ for **AZB-TRZ** in the 10 wt % doped mCP film. Finally, the Φ_PL_ of the films was found to vary within experimental error, in the moderate range of 30–40% for each material in the two OLED hosts (Table S3). For **AZB-TRZ**, the significant increase in film Φ_PL_ compared to toluene solution again supports the hypothesis that access to the bent conformer may act as a quenching pathway, but only in fluid media where conformational interconversion is feasible.

### Device Characterization

**Figure 8:**
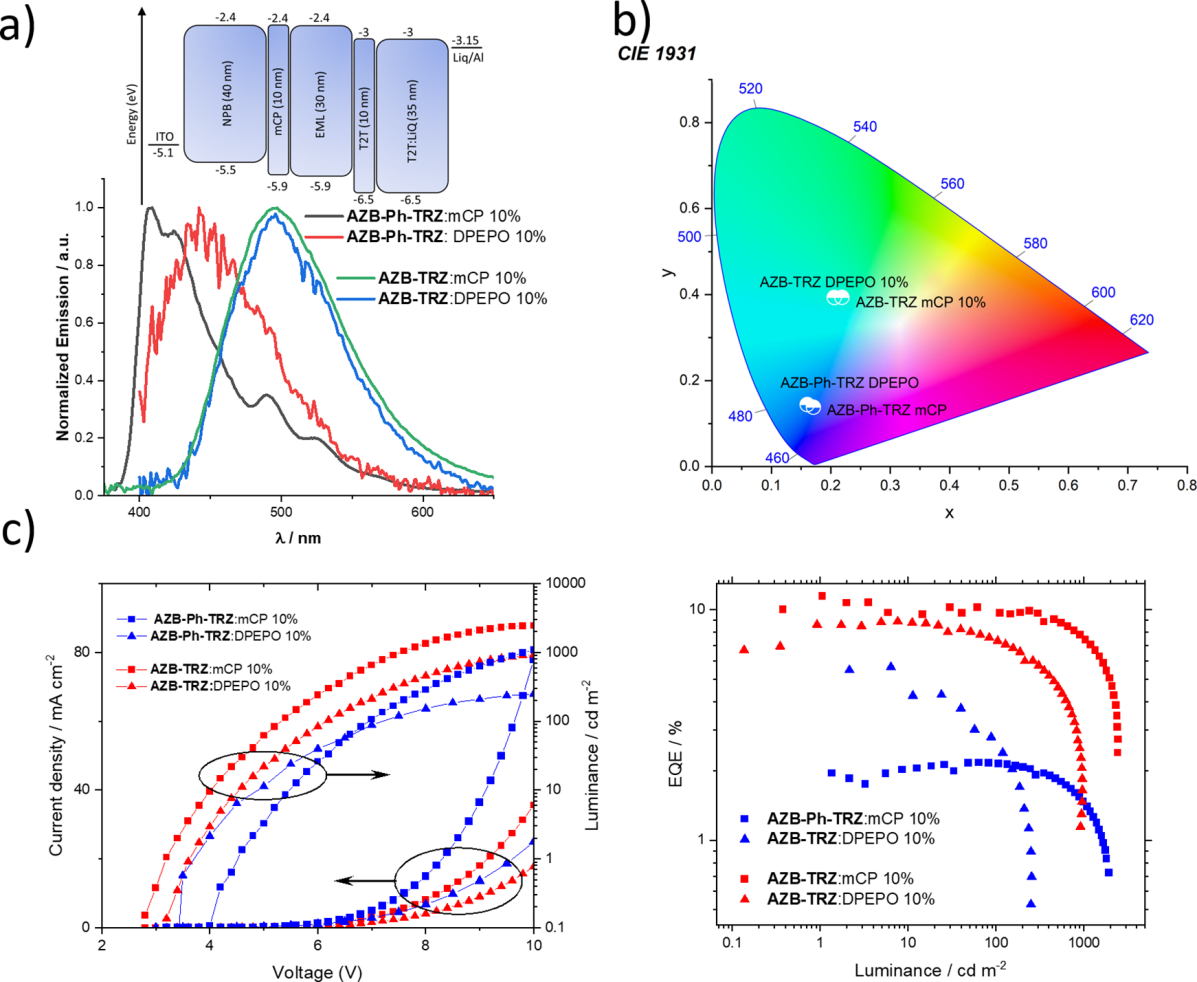
OLED performance of **AZB-Ph-TRZ** and **AZB-TRZ** in different hosts: (a) EL spectra and device structure; (b) CIE coordinates of device emission; (c) current and luminance at different voltages; (d) external quantum efficiency at different luminances.

Deployed into OLEDs using a structure of ITO (anode)|NPB (HIL/HTL, 40 nm)|mCP (EBL, 10 nm)|emitter:host 10% or 20% (EML, 30 nm)|T2T (HBL, 10 nm)|T2T:LiQ 50% (EIL/ETL, 35 nm)|LiQ (0.7 nm)|Al (cathode, 100 nm), the performance of the devices with **AZB-Ph-TRZ** and **AZB-TRZ** conforms broadly to the trends established by the optical results and Φ_PL_ values (Figure 8). Reflecting its stronger DF and faster *k*_RISC_, **AZB-TRZ** in mCP achieves a higher EQE_max_ of 10.5% and reduced efficiency roll-off compared to the device using DPEPO host. This EQE_max_ compares favorably to the equivalent film Φ_PL_ (34% in mCP), indicating highly efficient exciton harvesting in the emissive layer based on unassisted optical outcoupling. Increasing the emitter doping to 20 wt % from 10 wt % makes minimal difference to the electroluminescence (EL) spectrum (Figure S30); however, although the EQE_max_ is attenuated (6.3%), much higher luminance nearing 10000 cd m^–2^ can be attained. The device with **AZB-TRZ** in DPEPO exhibits very similar EL and the same performance trends as in mCP, although with a generally lower EQE_max_ (8.9%) and severe efficiency roll-off, limiting maximum brightness to just below 1000 cd m^–2^ (Figure 8d). These results in different hosts are consistent with their similar Φ_PL_ and worse DF emission intensity and kinetics in DPEPO.

The devices with **AZB-Ph-TRZ** show much lower EQE_max_ and more severe efficiency roll-off than those with **AZB-TRZ**. However, the performance of the devices using **AZB-Ph-TRZ** varies more significantly depending on the host, in terms of the EL spectrum, EQE_max_, and efficiency roll-off. Based on the shapes and positions of the EL spectra, we consider the mCP:**AZB-Ph-TRZ** device to be emitting from the ^1^LE state (as in toluene and Zeonex), which frustrates efficient triplet harvesting by RISC and leads to lower efficiency. Indeed, comparing the maximum efficiency (2.1%) with the film Φ_PL_ (27%) reveals that triplet harvesting is hardly active in these devices, consistent with the very large Δ*E*_ST_ of 0.39 eV in the mCP host. Conversely, in the DPEPO host the EL spectrum is red-shifted and broader, indicative of ^1^CT emission; this switchover is attributed to the larger polarity/polarizability of the DPEPO host.^49,50,52^ This difference in exciton character together with reduced Δ*E*_ST_ (0.15 eV) results in more efficient RISC and a higher EQE_max_ of 5.6% despite a lower film Φ_PL_ of 20%. The efficiency roll-off is still considerably strong in DPEPO though, a consequence of its well-documented electrical instability.^53,54^

## Conclusions

We have introduced azaborine as a very weak donor moiety for the construction of new D-A TADF emitters. As an electron donor AZB is weak like carbazole but due to its size and shape adopts a perpendicular conformation in D-A systems similar to DMAC-containing compounds. Together, these properties lead to blue-shifted PL while retaining TADF activity. This donor can also adopt a bent conformer that quenches emission, although this is suppressed in solid hosts. In the presented example materials, **AZB-TRZ** exhibits overall better TADF properties and OLED performance than those of **AZB-Ph-TRZ** because of its smaller Δ*E*_ST_ and good Φ_PL_. Yet greater performance in blue TADF materials may be discovered using this new donor in conjunction with other acceptors in the future.

## Experimental Section

### General Methods

The following starting materials were synthesized according to literature materials: bis(2-bromophenyl)amine^55^ and dimethyl mesitylboronate.^56^ Air-sensitive reactions were performed under a nitrogen atmosphere using Schlenk techniques; no special precautions were taken to exclude air or moisture during workup and crystallization. HPLC analysis was conducted on a Shimadzu Prominence Modular HPLC system. HPLC traces were performed using an ACE Excel 2 C18 analytical column. Melting points were measured using open-ended capillaries on an Electrothermal 1101D Mel-Temp apparatus and are uncorrected. High-resolution mass spectrometry (HRMS) was performed at the University of Edinburgh. Elemental analyses were performed by the School of Geosciences at the University of Edinburgh.

### 5-Benzyl-10-mesityl-5,10-dihydrodibenzo[*b*,*e*][1,4]azaborinine (**2**)

To a solution of *N*,*N*-bis(2-bromophenyl)benzylamine (2 g, 4.79 mmol, 1 equiv) in dry diethyl ether (50 mL) at −78 °C under a nitrogen atmosphere was portionwise added *n*-butyllithium (1.6 M in hexane, 10.07 mmol, 2.1 equiv). The resulting solution was stirred at rt for 30 min. To the solution was added dimethyl mesitylboronate (1.01 g, 5.27 mmol, 1.1 equiv), and the mixture was stirred at rt for 4 h. The reaction mixture was treated with aqueous NH_4_Cl and then extracted with DCM (3 × 25 mL). The combined organic layers were dried with anhydrous sodium sulfate and concentrated under reduced pressure. The crude mixture was purified by silica gel flash column chromatography using hexane:DCM = 3:7 as eluent to afford the desired compound as a white solid. Yield: 86%. *R*_f_: 0.46 (hexane:DCM = 2:1 on silica gel). Mp: 196–199 °C (crystals). ^1^H NMR (400 MHz, CDCl_3_): δ (ppm) 7.91 (d, *J* = 7.56 Hz, 2 H), 7.65 (t, *J* = 7.73 Hz, 2 H), 7.49 (d, *J* = 8.72 Hz, 2 H), 7.41–7.28 (m, 5 H), 7.15 (t, *J* = 6.92 Hz, 2 H), 6.99 (s, 2 H), 5.79 (s, 2 H), 2.43 (s, 3 H), 2.03 (s, 6 H). ^13^C NMR (125 MHz, CDCl_3_): δ (ppm) 146.3, 139.26, 137.48, 136.71, 136.38, 133.62, 129.09, 127.42, 126.81, 125.89, 119.91, 115.30, 52.71, 23.28, 21.31. GC-MS [M]^+^ Retention time: 13.24 min. Calculated: (C_28_H_26_BN) 387.21; Found: 387.25.

### 10-Mesityl-5,10-dihydrodibenzo[**b**,**e**][1,4]azaborinine (**AZB**)

To a solution of 5-benzyl-10-mesityl-5,10-dihydrodibenzo[*b*,*e*][1,4]azaborinine (387 mg, 1 mmol, 1 equiv) in mixed solvent of dichloromethane (40 mL). ethanol (30 mL). and ethyl acetate (20 mL) was added Pd on charcoal (400 mg, 10%(w/w), 0.37 mmol of Pd, 0.37 equiv), and the resulting mixture was stirred at room temperature in the presence of hydrogen gas for 2 days. The reaction mixture was passed through a Celite bed and washed with dichloromethane. The reaction mixture was then concentrated in vacuo and purified by column chromatography on silica gel using hexane:DCM = 2:3 as eluent to afford the desired compound as a white solid. The unreacted material was recovered. Yield: 20%. *R*_f_: 0.52 (hexane:DCM = 1:1 on silica gel). Mp: 217–219 °C (crystals). Lit.: 230–233 °C.^57^^1^H NMR (400 MHz, CDCl_3_): δ (ppm) 8.43 (s, 1 H), 7.82 (d, *J* = 7.56 Hz, 2 H), 7.67 (t, *J* = 7.60 Hz, 2 H), 7.49 (d, *J* = 8.28 Hz, 2 H), 7.11 (t, *J* = 7.40 Hz, 2 H), 2.40 (s, 3 H), 1.98 (s, 6 H). ^13^C NMR (125 MHz, CDCl_3_): δ (ppm) 143.64, 139.24, 136.89, 136.38, 133.07, 126.83, 119.88, 116.13, 23.15, 21.34. The ^1^H and ^13^C NMR spectra match those in the literature.^57^

### General Procedure for the Synthesis of Azaborine-Based Emitters

An oven-dried Schlenk flask held under a nitrogen atmosphere was charged with dry toluene (50 mL), 10-mesityl-5,10-dihydrodibenzo[*b*,*e*][1,4]azaborinine (1 equiv), triazine derivative (1.2 equiv), XPhos (0.32 equiv), palladium acetate (0.22 equiv), and sodium *tert*-butoxide (3 equiv). The reaction mixture was then heated at 100 °C for 12 h. After cooling, the mixture was passed through a Celite pad and concentrated in vacuo. The combined organic layer was dried with anhydrous sodium sulfate and concentrated in vacuo. The resulting mixture was purified by silica gel column chromatography to yield the desired compound.

#### 5-(4-(4,6-Diphenyl-1,3,5-triazin-2-yl)phenyl)-10-mesityl-5,10-dihydrodibenzo[*b*,*e*][1,4]azaborinine (**AZB-Ph-TRZ**)

The quantities used for the reaction are as follows: 10-mesityl-5,10-dihydrodibenzo[b,e][1,4]azaborinine (500 mg, 1.68 mmol, 1 equiv), 2-(4-bromophenyl)-4,6-diphenyl-1,3,5-triazine (784 mg, 2.02 mmol, 1.2 equiv), XPhos (257 mg, 0.54 mmol, 0.32 equiv), palladium acetate (83 mg, 0.37 mmol, 0.22 equiv), NaO^*t*^Bu (485 mg, 5.05 mmol, 3 equiv). White solid. Yield: 70%. Mp: 314–317 °C. *R*_f_: 0.26 (hexane:DCM = 2:1, silica gel). The target compound was then purified by silica gel column chromatography (hexane:DCM = 4:1, silica gel). ^1^H NMR (400 MHz, CDCl_3_): δ (ppm) 9.15 (d, *J* = 8.36 Hz, 2 H), 8.86 (d, *J* = 7.60 Hz, 2H), 7.93 (d, *J* = 7.60 Hz, 2H), 7.69–7.60 (m, 8H), 7.54 (t, *J* = 7.60 Hz, 2H), 7.15 (t, *J* = 7.60 Hz, 2H), 7.01 (s, 2H), 6.96 (d, *J* = 8.72 Hz, 2H), 2.44 (s, 3H), 2.08 (s, 6H). ^13^C NMR (125 MHz, CDCl_3_): δ (ppm) 172, 170.9, 146.3, 145.4, 139.4, 137.2, 137, 136.5, 136, 132.9, 132.8, 131.6, 130.9, 129.1, 128.8, 126.9, 126, 119.9, 116.9, 23.3, 21.4. HR-MS[M + H]^**+**^ Calculated: (C_42_H_34_B_1_N_4_) 605.2871; Found: 605.2849. Anal. Calcd for C_36_H_24_N_6_O_2_: C, 83.44%; H, 5.50%; N, 9.27%. Found: C, 84.02%; H, 5.66%; N, 9.21%. HPLC: 100%, retention time 6.72 min in 98% MeCN/2% H_2_O.

#### 5-(4,6-Diphenyl-1,3,5-triazin-2-yl)-10-mesityl-5,10-dihydrodibenzo[*b*,*e*][1,4]azaborinine (**AZB-TRZ**)

The quantities used for the reaction are as follows: 10-mesityl-5,10-dihydrodibenzo[*b*,*e*][1,4]azaborinine (750 mg, 2.52 mmol, 1 equiv), 2-chloro-4,6-diphenyl-1,3,5-triazine (811 mg, 3.03 mmol, 1.2 equiv), XPhos (385 mg, 0.81 mmol, 0.32 equiv), palladium acetate (124 mg, 0.55 mmol, 0.22 equiv), NaO^*t*^Bu (727 mg, 7.57 mmol, 3 equiv). White solid. Yield: 53%. Mp: 249–251 °C. *R*_f_: 0.56 (hexane:DCM = 1:1, silica gel). The target compound was then purified by silica gel column chromatography (hexane:DCM = 3:2, silica gel). ^1^H NMR (400 MHz, CDCl_3_): δ (ppm) 8.76–8.74 (m, 4H), 7.93 (d, *J* = 7.60 Hz, 2H), 7.66 (t, *J* = 7.32 Hz, 2H), 7.59–7.52 (m, 6H), 7.17 (t, *J* = 7.35 Hz, 2H), 7 (s, 2H), 6.93 (d, *J* = 8.64 Hz, 2H), 2.44 (s, 3H), 2.09 (s, 6H). ^13^C NMR (125 MHz, CDCl_3_): δ (ppm) 176.1, 169.7, 144.4, 139.4, 137.3, 136.6, 134.9, 133.7, 133.2, 129.6, 129, 126.9, 125.8, 120.7, 115.8, 23.3, 21.4. HR-MS[M + H]^**+**^ Calculated: (C_36_H_30_BN_4_) 529.2558; Found: 529.2556. Anal. Calcd for C_36_H_29_BN_4_: C, 81.82%; H, 5.53%; N, 10.60. Found: C, 81.91; H, 5.73; N, 10.42. HPLC: 98%, retention time: 6.84 min in 90% MeCN/10% H_2_O.

### Theoretical Calculations

All ground-state optimizations were carried out at the density functional theory (DFT) level with Gaussian16^58^ using the PBE0 functional and the 6-31G(d,p) basis set. Excited-state calculations have been performed at time-dependent DFT (TD-DFT) within the Tamm–Dancoff approximation (TDA)^59,60^ using the same functional and basis set as for ground state geometry optimization. Spin–orbit coupling matrix elements (ξ) were calculated based on the optimized triplet excited state geometry. Molecular orbitals were visualized using GaussView 6.0.^61^ Calculations were automated using an in-house designed software package, Silico, which uses a number of third party libraries and programs, including extraction and processing of results cclib,^62^ generations of 3D images VMD^63^ and Tachyon.^64^

### Electrochemistry Measurements

Cyclic voltammetry (CV) analysis was performed on an electrochemical analyzer potentiostat, Model 620E from CH Instruments, at a sweep rate of 100 mV/s. Differential pulse voltammetry (DPV) was conducted with an increment potential of 0.004 V and pulse amplitude, width, and period of 50 mV, 0.05, and 0.5 s, respectively. Samples were prepared as DCM solutions, which were degassed by sparging with MeCN-saturated argon gas for 5 min prior to measurements. All measurements were performed using a 0.1 M DCM solution of tetra-*n*-butylammonium hexafluorophosphate ([^*n*^Bu_4_N]PF_6_]). An Ag/Ag^+^ electrode was used as the reference electrode, while a platinum electrode and a platinum wire were used as the working electrode and counter electrode, respectively. The redox potentials are reported relative to a saturated calomel electrode (SCE) with a ferrocenium/ferrocene (Fc/Fc^+^) redox couple as the internal standard (0.46 V vs SCE).^65^

### Photophysical Measurements

Optically dilute solutions of concentrations on the order of 10^–5^ or 10^–6^ M were prepared in spectroscopic or HPLC grade solvents for absorption and emission analysis. Absorption spectra were recorded at room temperature on a Shimadzu UV-2600 double-beam spectrophotometer with a 1 cm quartz cuvette. Molar absorptivity determination was verified by a linear regression analysis of values obtained from at least four independent solutions at varying concentrations with absorbance ranging from 0.036 to 0.173 for **AZB-Ph-TRZ** and from 0.034 to 0.146 for **AZB-TRZ**.

For emission studies, aerated solutions were bubbled by compressed air for 5 min and spectra were taken using the cuvette for absorption analysis. Degassed solutions were prepared via three freeze–pump–thaw cycles, and spectra were taken using a homemade Schlenk quartz cuvette. Steady-state emission, excitation, and time-resolved emission spectra were recorded at 298 K using an Edinburgh Instruments FS5 instrument. Samples were excited at 340 nm for steady-state measurements and at 375 nm for time-resolved measurements. Photoluminescence quantum yields for solutions were determined using the optically dilute method in which four sample solutions with absorbances of ca. 0.11, 0.075, 0.054, and 0.029 at 358 nm were used. The Beer–Lambert law was found to remain linear at the concentrations of the solutions. For each sample, linearity between absorption and emission intensity was verified through a linear regression analysis with the Pearson regression factor (*R*^2^) for the linear fit of the data set surpassing 0.9. Individual relative quantum yield values were calculated for each solution, and the values reported represent the slope obtained from the linear fit of these results. The quantum yield of the sample, Φ_PL_, can be determined by the equation (66) where *A* stands for the absorbance at the excitation wavelength (λ_exc_ = 358 nm), *I* is the integrated area under the corrected emission curve, and *n* is the refractive index of the solvent with the subscripts “s” and “r” representing sample and reference, respectively. Φ_r_ is the absolute quantum yield of the external reference quinine sulfate (Φ_r_ = 54.6% in 1 *N* H_2_SO_4_).^67^ The experimental uncertainty in the emission quantum yields is conservatively estimated to be 10%, though we have found that statistically we can reproduce Φ_PL_s to 3% relative error.

To prepare the 10 wt % doped films of emitters in a host matrix, 90% w/w (90 mg) of the host was dissolved in 1 mL of solvent and to this was added 10% w/w (10 mg) of emitter. Thin films were then spin-coated on a quartz substrate using a spin speed of 1500 rpm for 60 s. An integrating sphere (FS5) was employed for quantum yield measurements for thin film samples. The steady-state fluorescence of the doped solid-state films was measured using a Horiba Jobin Yvon Fluorolog-3 spectrofluorometer. Time-resolved measurements were performed using a spectrograph (Horiba Triax) and a Stanford Computer Optics 4Picos ICCD camera, where samples were excited with a Nd:YAG laser (EKSPLA, 10 Hz, 355 nm) either under vacuum at room temperature or under a stream of dry temperature-controlled nitrogen gas (Janis VNF-100 cryostat).

### OLED Fabrication and Testing

OLEDs were fabricated on patterned indium tin oxide (ITO) coated glass (VisionTek Systems) with a sheet resistance of 15 Ω/sq. Oxygen-plasma-cleaned substrates were loaded into a Kurt J. Lesker Super Spectros deposition chamber, and both the small molecule and cathode layers were thermally evaporated at a pressure of below 10^–7^ mbar. The materials used for the transport and blocking layers were *N*,*N*-bis(naphthalen-1-yl)-*N*,*N*-bis(phenyl)benzidine (NPB) as the hole injection/transport layer (HIL/HTL), 1,3-di(9*H*-carbazol-9-yl)benzene, *N*,*N*′-dicarbazolyl-3,5-benzene (mCP) as the electron-blocking layer (EBL), the emissive layer (EML) had mCP or DPEPO as a host doped with the TADF emitter, 2,4,6-tris(biphenyl-3-yl)-1,3,5-triazine (T2T) as the hole-blocking layer (HBL), T2T and 8-hydroxyquinolinolato-lithium (Liq) as the electron transport/injection layer (ETL/EIL), and an aluminum (Al) cathode. NPB, mCP, and T2T were purchased from Sigma-Aldrich and sublimed before use.

Freshly evaporated devices were transferred into either a calibrated 6 in. integrating sphere in a glovebox or a calibrated 10 in. sphere under ambient conditions. Electrical properties were measured using a source meter (Keithley 2400) simultaneously with emission spectrum and intensity with a calibrated fiber coupled spectrometer (Ocean optics USB4000). In the 6 in. sphere an additional silicon photodiode was used to monitor very low luminance. All devices were evaluated at 293 K.

## Data Availability

The research data supporting this publication can be accessed at 10.17630/e2d1fc63-f102-44ef-ba9c-10a168e9fd92.

## Supporting Information Available

^1^H and ^13^C NMR spectra, HRMS, HPLC, EA, and single-crystal XRD structures (CCDC: 2219210–2219212) of the target compounds, additional film spectra and decays, and OLED comparisons at higher loading (PDF)
Optimized geometry coordinates (TXT)
