# Supplementary material for: Azaborine as a Versatile Weak Donor for Thermally Activated Delayed Fluorescence
Source: ACS Appl Mater Interfaces. 2023 May 18;15(21):25806–18. doi: 10.1021/acsami.3c05409 (PMC10236432; doi:10.1021/acsami.3c05409)
Supplement: Supplementary file 1 — am3c05409_si_001.pdf [file am3c05409_si_001.pdf]

Supporting Information

Azaborine as a Versatile Weak Donor for Thermally Activated Delayed Fluorescence

Pagidi Sudhakar,[a+] Suman Kuila,[b+] Kleitos Stavrou,[b] Andrew Danos,[b] Alexandra M. Z. Slawin,[a] Andrew Monkman,[b] and Eli Zysman-Colman[a]**

[a]Organic Semiconductor Centre, EaStCHEM School of Chemistry, University of St Andrews,

St Andrews, UK, KY16 9ST. E-mail: eli.zysman-colman@st-andrews.ac.uk

[b]Department of Physics, Durham University, Durham, DH1 3LE, UK.

E-mail: andrew.danos@durham.ac.uk

Table of contents

Section	Pages
General methods	S3
Synthesis and chemical characterization (NMR, HRMS and X-ray)	S4
DFT results	S17
Optical measurements data	S18
Device characterization	S25

Experimental Section

General Methods. All other reagents and solvents were obtained from commercial sources and used as received. Anhydrous THF, DCM, toluene and diethyl ether was/were obtained from a MBraun SPS5 solvent purification system. Flash column chromatography was carried out using silica gel (Silia-P from Silicycle, 60 Å, 40-63 μm). Analytical thin-layer-chromatography (TLC) was performed with silica plates with aluminum backings (250 μm with F-254 indicator). TLC visualization was accomplished by 254/365 nm UV lamp. HPLC analysis was conducted on a Shimadzu LC-40 HPLC system. GCMS analysis was conducted using a Shimadzu QP2010SE GC-MS equipped with a Shimadzu SH-Rtx-1 column (30 m × 0.25 mm). ^{1}H, and ^{13}C spectra were recorded on a Bruker Advance spectrometer (400 MHz for ^{1}H, 125 MHz for ^{13}C). The following abbreviations have been used for multiplicity assignments: "s" for singlet, "d" for doublet, "t" for triplet and "m" for multiplet. ^{1}H and ^{13}C NMR spectra were referenced residual solvent peaks with respect to TMS (δ = 0 ppm).

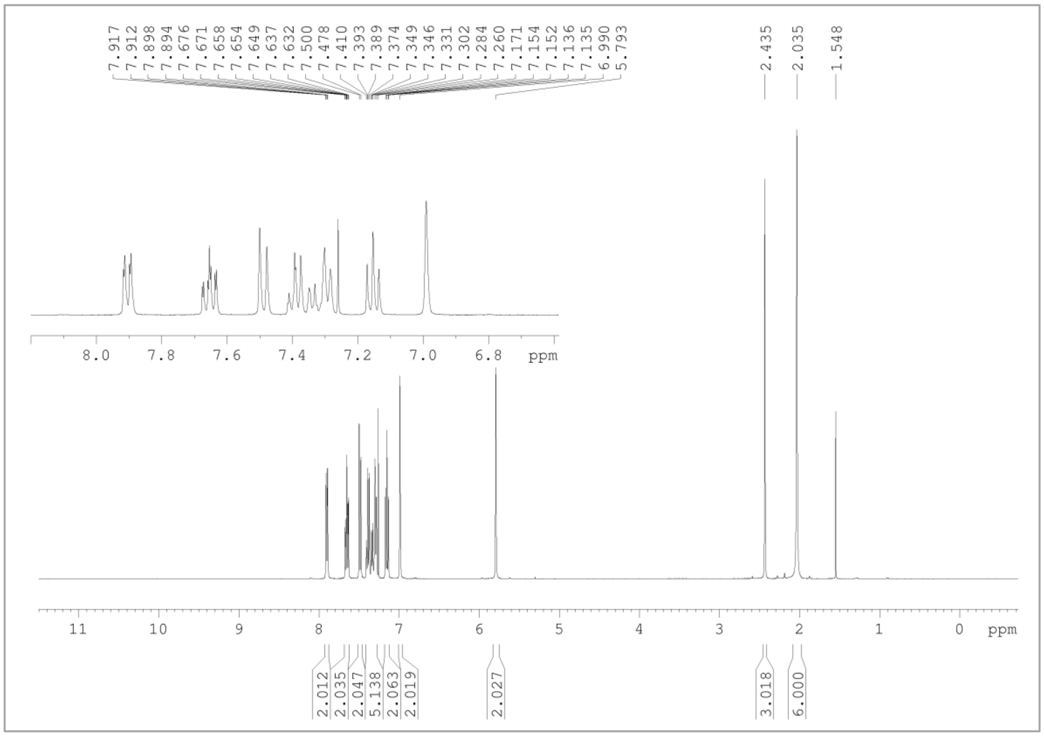

Figure **S1**. ^{1}H NMR of **2** in $CDCl_3$

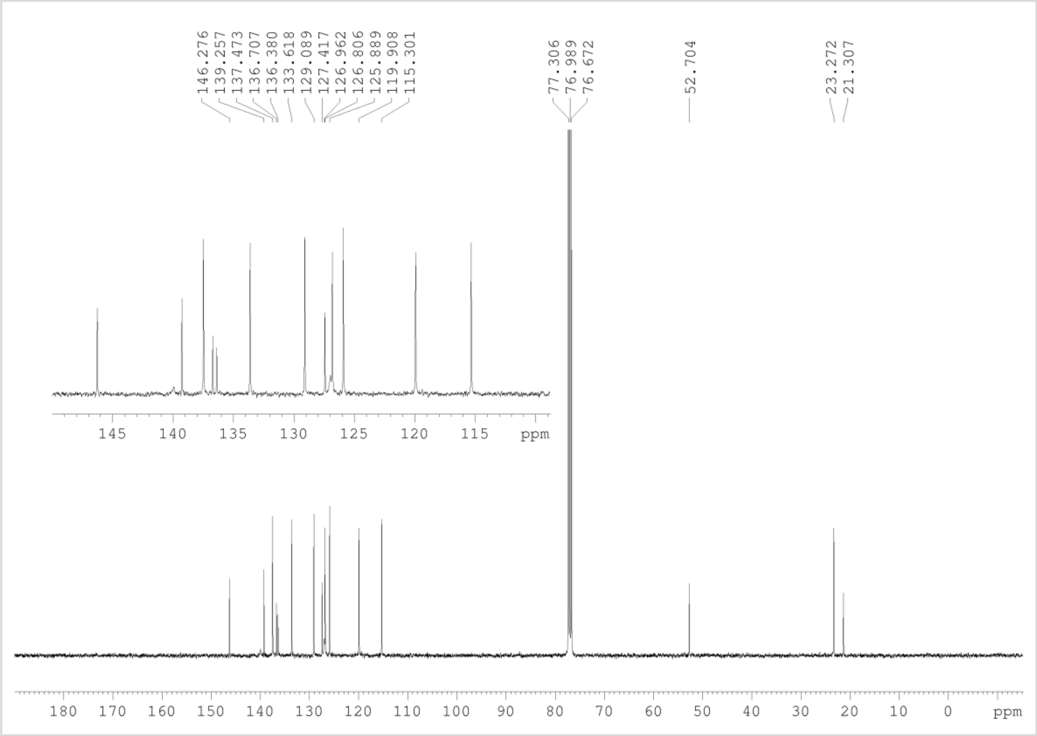

Figure **S2**. ^{13}C NMR of **2** in $CDCl_3$

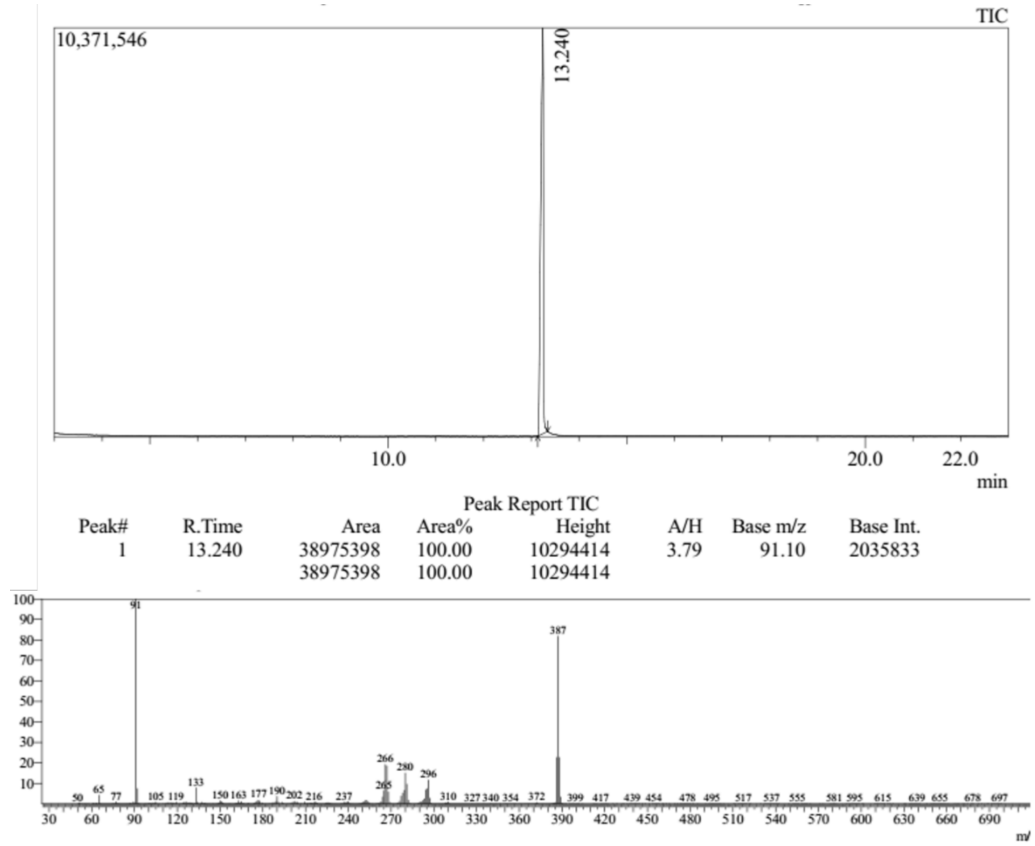

Peak Report TIC

Peak#	R.Time	Area	Area%	Height	A/H	Base m/z	Base Int.
1	13.240	38975398	100.00	10294414	3.79	91.10	2035833
		38975398	100.00	10294414			

Figure **S3**. GCMS of compound **2**

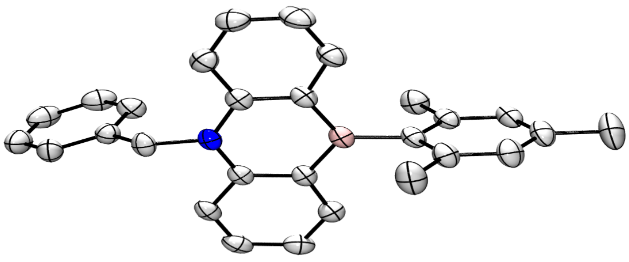

Figure **S4**. ORTEP molecular structure of **2** determined from X-ray diffraction (displacement parameters are drawn at 50% probability level and H-atoms omitted for clarity).

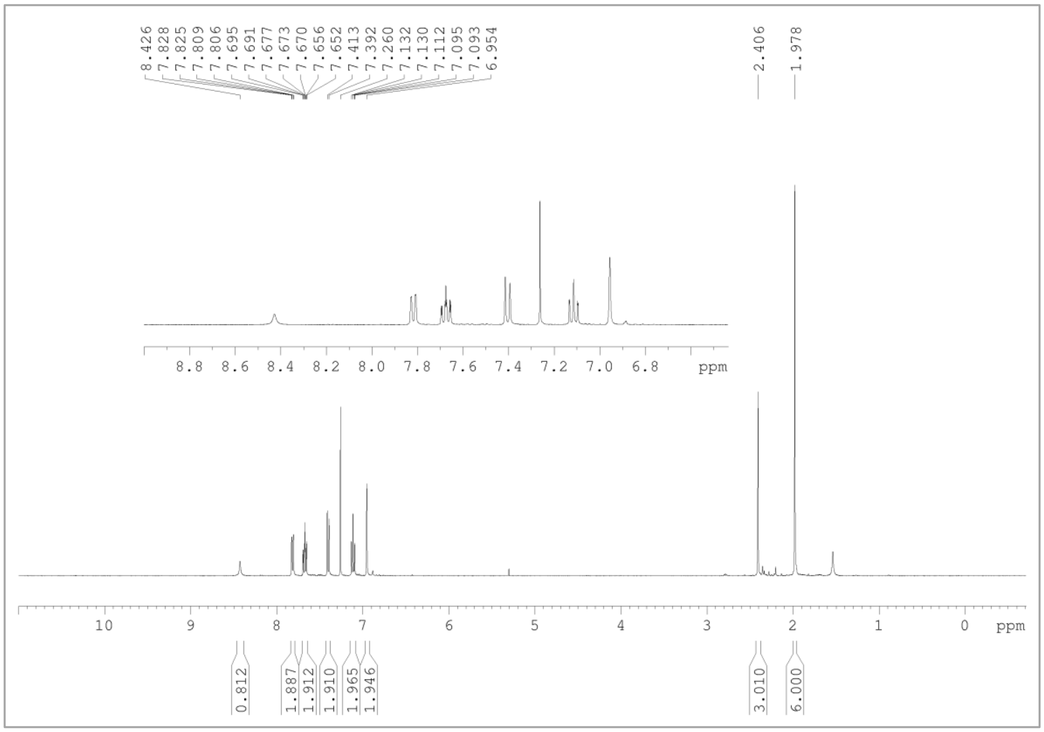

Figure **S5**. ^{1}H NMR of **AZB** in $CDCl_3$

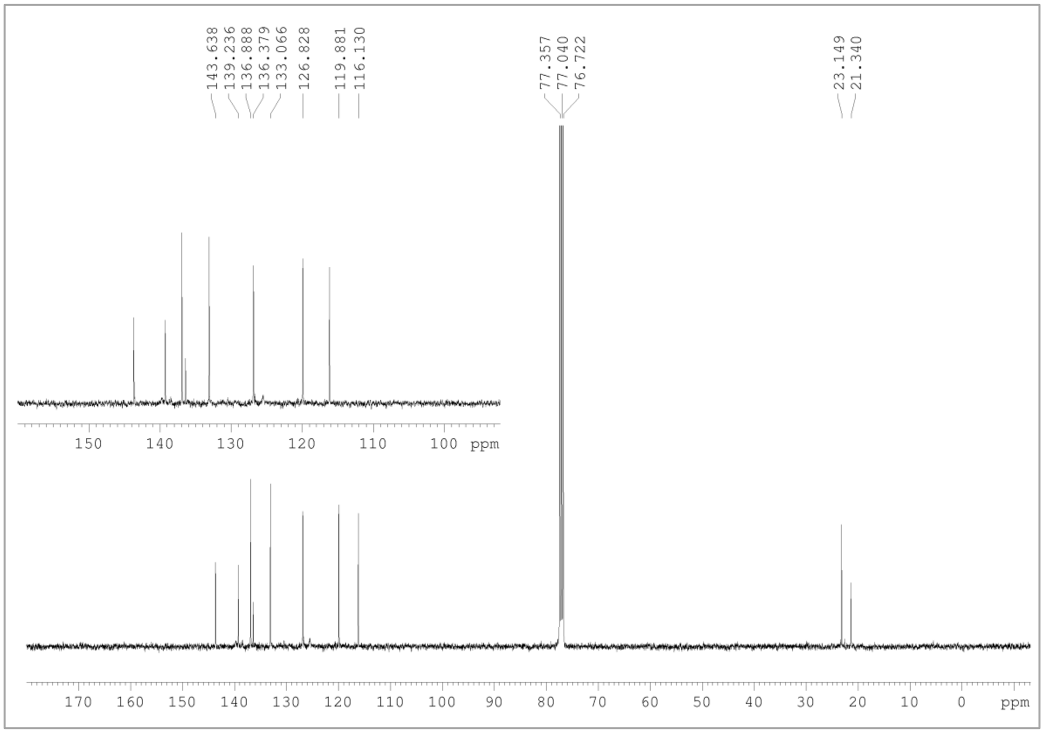

Figure **S6**. ^{13}C NMR of **AZB** in $CDCl_3$

AZB-Ph-TRZ:

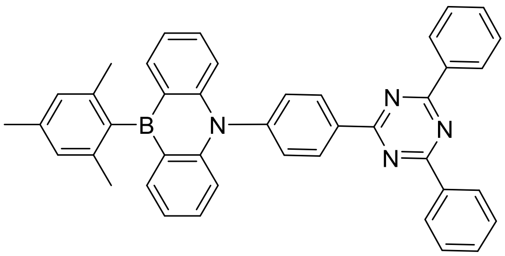

The quantities used for the reaction are as follows. 10-mesityl-5,10-dihydrodibenzo[b,e][1,4]azaborinine (500 mg, 1.68 mmol, 1 equiv.), 2-(4-bromophenyl)-4,6-diphenyl-1,3,5-triazine (784 mg, 2.02 mmol, 1.2 equiv.), XPhos (257 mg, 0.54 mmol, 0.32 equiv.), palladium acetate (83 mg, 0.37 mmol, 0.22 equiv.), NaO^{t}Bu (485 mg, 5.05 mmol, 3 equiv.) White solid. **Yield:** 70%. **Mp:** 314-317 °C. **R$_f$:** 0.26 (hexane : DCM = 2:1, silica gel). The target compound was then purified by Silica Gel Column Chromatography (hexane : DCM = 4:1, silica gel). (**^{1}H NMR (400 MHz, $CDCl_3$) δ (ppm)**: 9.15 (d, *J* = 8.36 Hz, 2 H), 8.86 (d, *J* = 7.60 Hz, 2H), 7.93 (d, *J* = 7.60 Hz, 2H), 7.69-7.60 (m, 8H), 7.54 (t, *J* = 7.60 Hz, 2H), 7.15 (t, *J* = 7.60 Hz, 2H), 7.01 (s, 2H), 6.96 (d, *J* = 8.72 Hz, 2H), 2.44 (s, 3H), 2.08 (s, 6H). **^{13}C NMR (125 MHz, $CDCl_3$) δ (ppm)**:172, 170.9, 146.3, 145.4, 139.4, 137.2, 137, 136.5, 136, 132.9, 132.8, 131.6, 130.9, 129.1, 128.8, 126.9, 126, 119.9, 116.9, 23.3, 21.4. **HR-MS[M+H]$^+$ Calculated: (**$C_{42}H_{34}B_1N_4$) 605.2871; **Found**: 605.2849. **Anal**. Calcd. for $C_{36}H_{24}N_6O_2$: C, 83.44%; H, 5.50%; N, 9.27%. Found: C, 84.02%; H, 5.66%; N, 9.21%. **HPLC**: 100%, retention time: 6.72 minutes in 98% MeCN/2% H_2O.

HPLC Trace Report28Jan2022

<Sample Information>

Sample Name	: SP-2933-BATCH1-FR1		
Sample ID	: 003		
Method Filename	: 98% Acetonitrile 2 Water 20 mins.lcm		
Batch Filename	: SP-2933.lcb		
Vial #	: 1-44	Sample Type	: Unknown
Injection Volume	: 5 uL		
Date Acquired	: 30/11/2021 15:04:09	Acquired by	: System Administrator
Date Processed	: 30/11/2021 15:24:11	Processed by	: System Administrator

<Chromatogram>

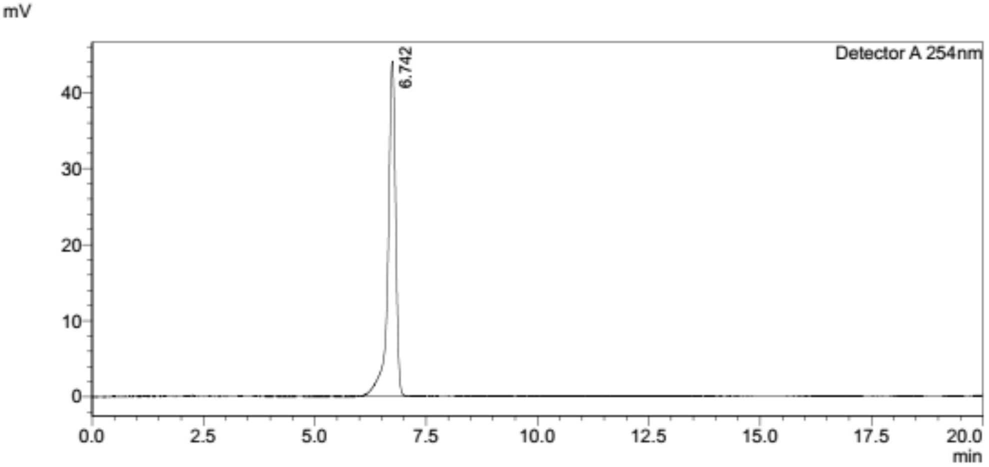

<Peak Table>

Detector A 254nm

Peak#	Ret. Time	Area	Height	Area%	Area/Height	Width at 5% Height
1	6.742	525527	43933	100.000	11.962	0.512
Total		525527	43933	100.000		

Figure **S7**. HPLC trace report of **AZB-Ph-TRZ**

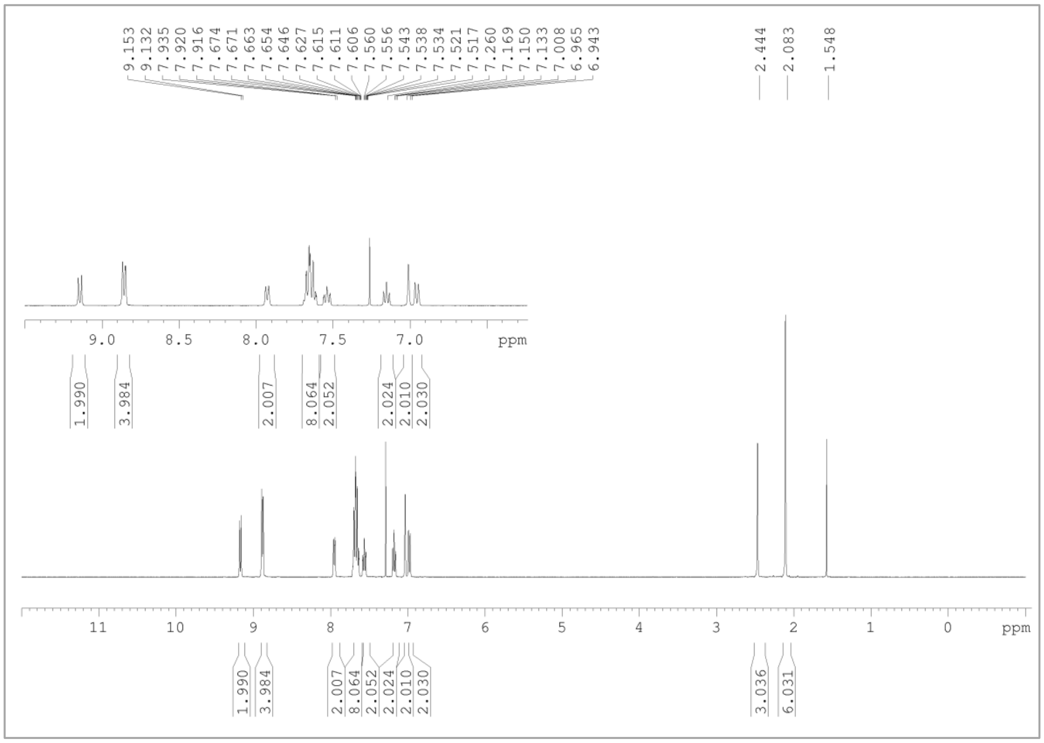

Figure **S8**. ^{1}H NMR of **AZB-Ph-TRZ** in $CDCl_3$

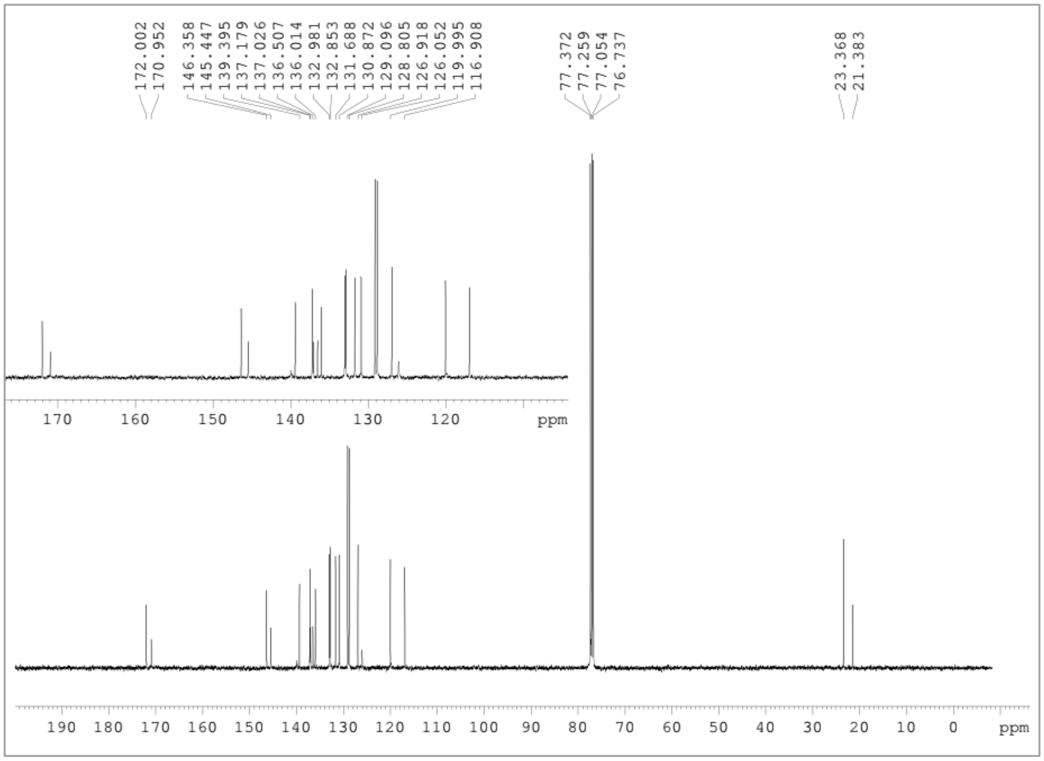

Figure **S9**. ^{13}C NMR of **AZB-Ph-TRZ** in $CDCl_3$

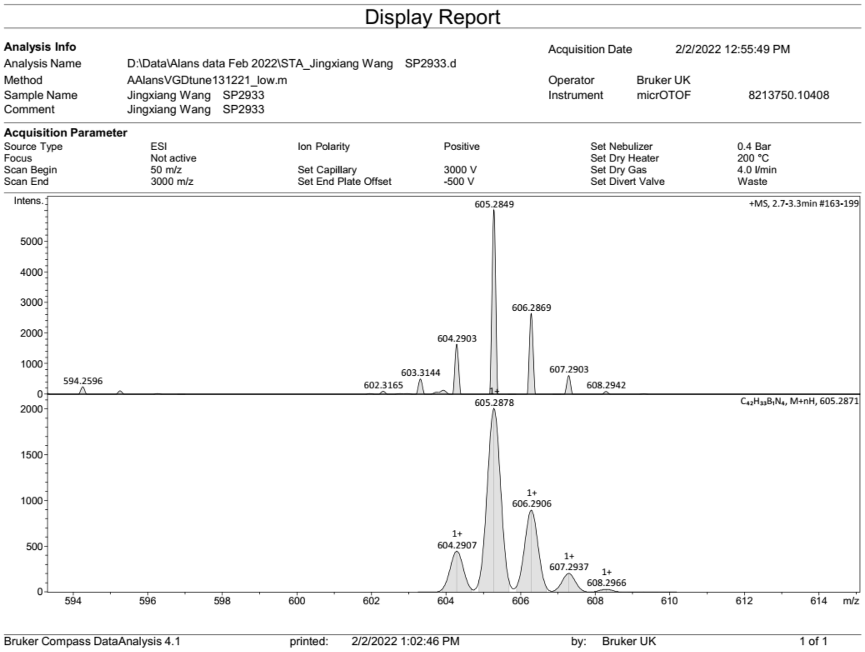

Figure **S10**. HRMS of **AZB-Ph-TRZ**

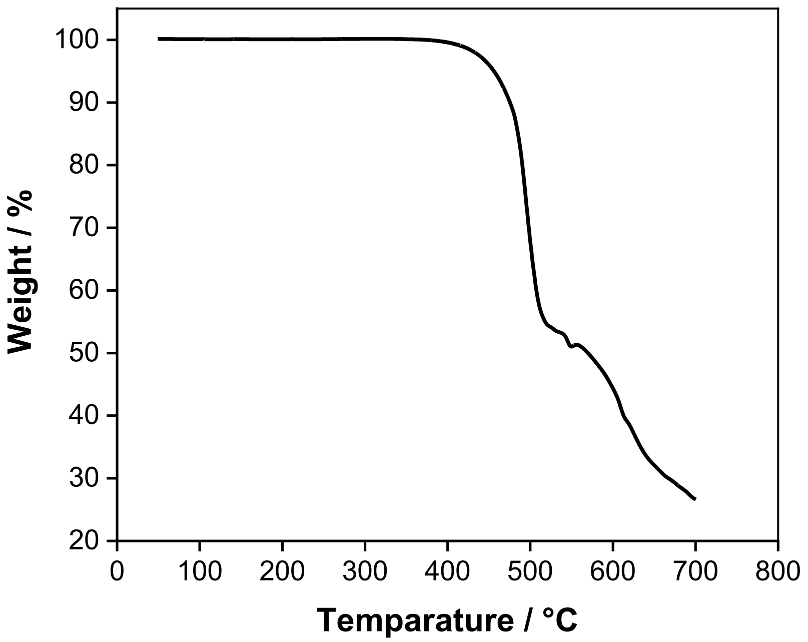

Figure **S11**. Thermogravimetric analysis curve of **AZB-Ph-TRZ**

AZB-TRZ:

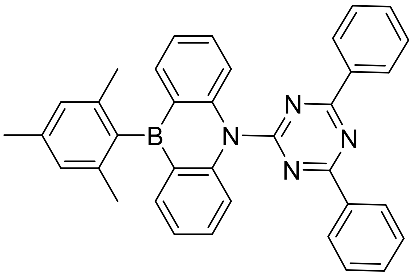

The quantities used for the reaction are as follows. 10-mesityl-5,10-dihydrodibenzo[b,e][1,4]azaborinine (750 mg, 2.52 mmol, 1 equiv.), 2-chloro-4,6-diphenyl-1,3,5-triazine (811 mg, 3.03 mmol, 1.2 equiv.), XPhos (385 mg, 0.81 mmol, 0.32 equiv.), palladium acetate (124 mg, 0.55 mmol, 0.22 equiv.), NaO^{t}Bu (727 mg, 7.57 mmol, 3 equiv.) White solid. **Yield:** 53%. **Mp:** 249**-** 251 °C. **R*f*:** 0.56 (hexane : DCM = 1:1, silica gel). The target compound was then purified by Silica Gel Column Chromatography again (hexane : DCM = 3:2, silica gel). (**^{1}H NMR (400 MHz, $CDCl_3$) δ (ppm)**: 8.76-8.74 (m, 4H), 7.93 (d, *J* = 7.60 Hz, 2H), 7.66 (t, *J* = 7.32 Hz, 2H), 7.59-7.52 (m, 6H), 7.17 (t, *J* = 7.35 Hz, 2H), 7 (s, 2H), 6.93 (d, *J* = 8.64 Hz, 2H), 2.44 (s, 3H), 2.09 (s, 6H). **^{13}C NMR (125 MHz, $CDCl_3$) δ (ppm)**: 176.1, 169.7, 144.4, 139.4, 137.3, 136.6, 134.9, 133.7, 133.2, 129.6, 129, 126.9, 125.8, 120.7, 115.8, 23.3, 21.4. **HR-MS[M+H]$^+$ Calculated:** ($C_{36}H_{30}BN_4$) 529.2558; **Found:** 529.2556. **Anal. Calcd. for $C_{36}H_{29}BN_4$:** C, 81.82%; H, 5.53%; N, 10.60. **Found**: C, 81.91; H, 5.73; N, 10.42. HPLC: 98%, retention time: 6.84 minutes in 90% MeCN/10% H_2O.

HPLC Trace Report31Aug2022

<Sample Information>

Sample Name : 3098
Sample ID :
Method Filename : 90% Acetonitrile 10 Water 20 mins.lcm
Batch Filename : 3098.lcb
Vial # : 1-52 Sample Type : Unknown
Injection Volume : 10 uL
Date Acquired : 22/01/2022 18:09:57 Acquired by : System Administrator
Date Processed : 22/01/2022 18:22:10 Processed by : System Administrator

<Chromatogram>

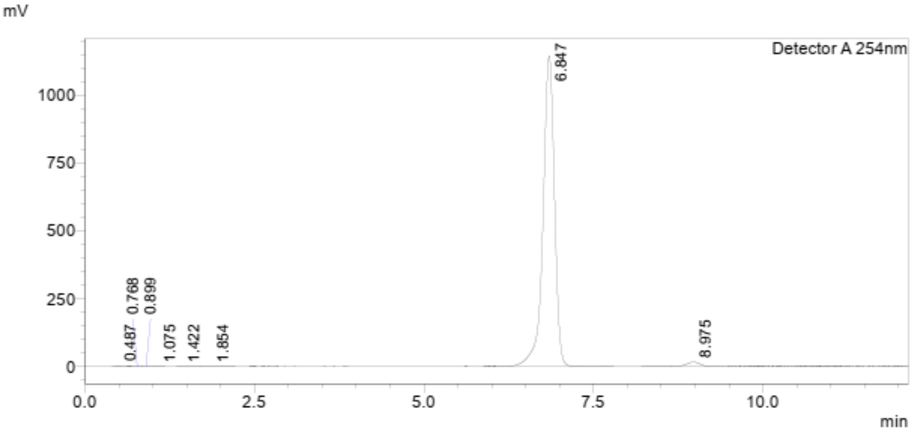

<Peak Table>

Detector A 254nm

Peak#	Ret. Time	Area	Height	Area%	Area/Height	Width at 5% Height
1	0.487	10108	2743	0.073	3.685	0.159
2	0.768	1846	452	0.013	4.085	--
3	0.899	2765	263	0.020	10.498	--
4	1.075	1702	207	0.012	8.227	--
5	1.422	3823	302	0.028	12.670	--
6	1.854	5048	499	0.037	10.125	--
7	6.847	13500319	1144475	98.079	11.796	0.439
8	8.975	239160	16214	1.737	14.750	0.551
Total		13764771	1165155	100.000		

Figure **S12**. HPLC trace report of **AZB-TRZ**

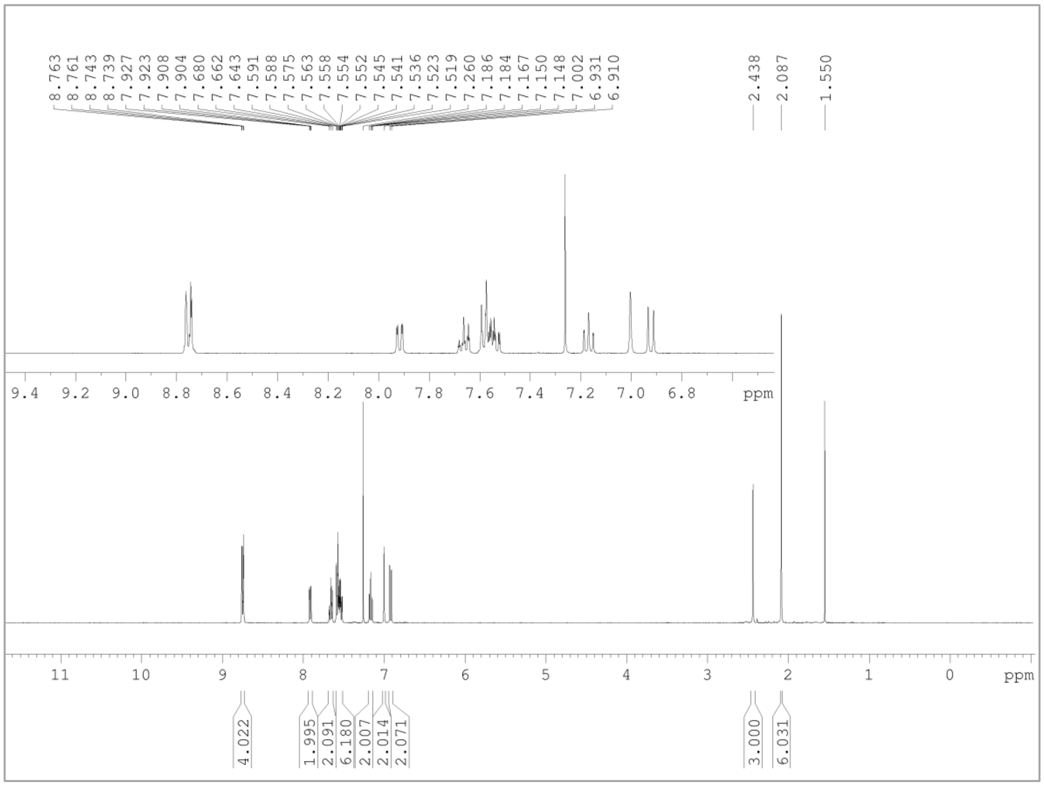

Figure **S13**. ^{1}H NMR of **AZB-TRZ** in $CDCl_3$

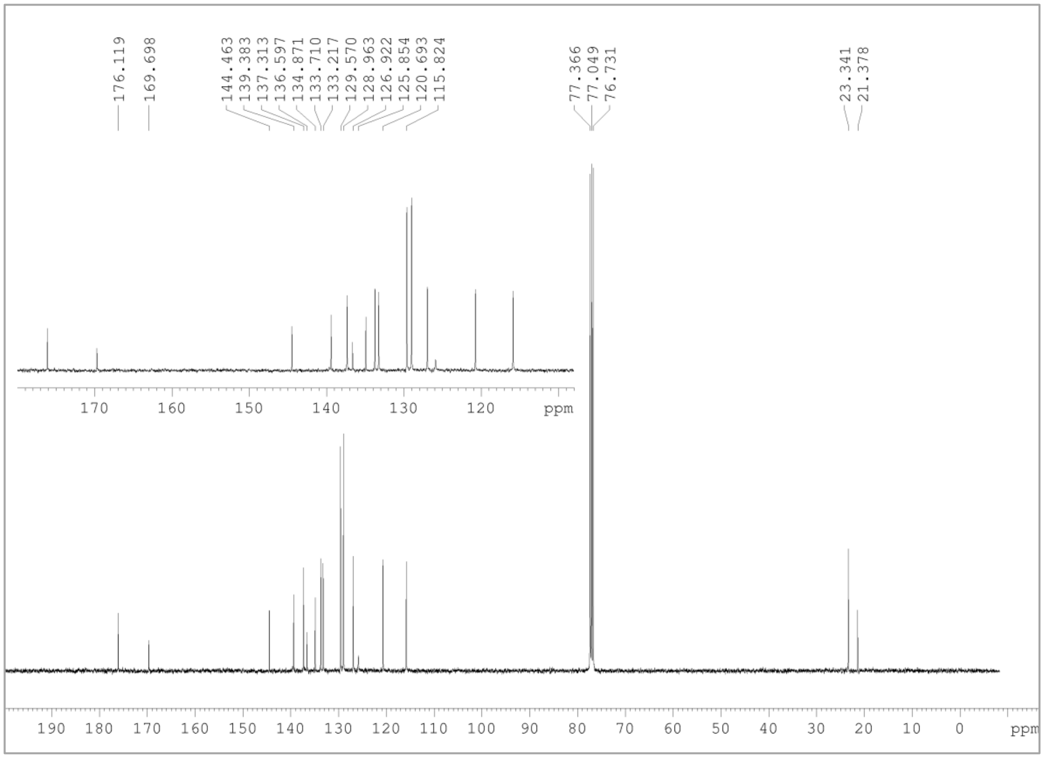

Figure **S14**. ^{13}C NMR of **AZB-TRZ** in $CDCl_3$

Display Report

Analysis Info

Analysis Name	D:\Data\Alans data Feb 2022\STA_Jingxiang Wang SP3098.d	Acquisition Date	2/2/2022 10:55:15 AM
Method	AAlansVGDtune131221_low.m	Operator	Bruker UK
Sample Name	Jingxiang Wang SP3098	Instrument	micrOTOF 8213750.10408
Comment	Jingxiang Wang SP3098		

Acquisition Parameter

Source Type	ESI	Ion Polarity	Positive	Set Nebulizer	0.4 Bar
Focus	Not active			Set Dry Heater	200 °C
Scan Begin	50 m/z	Set Capillary	3000 V	Set Dry Gas	4.0 l/min
Scan End	3000 m/z	Set End Plate Offset	-500 V	Set Divert Valve	Waste

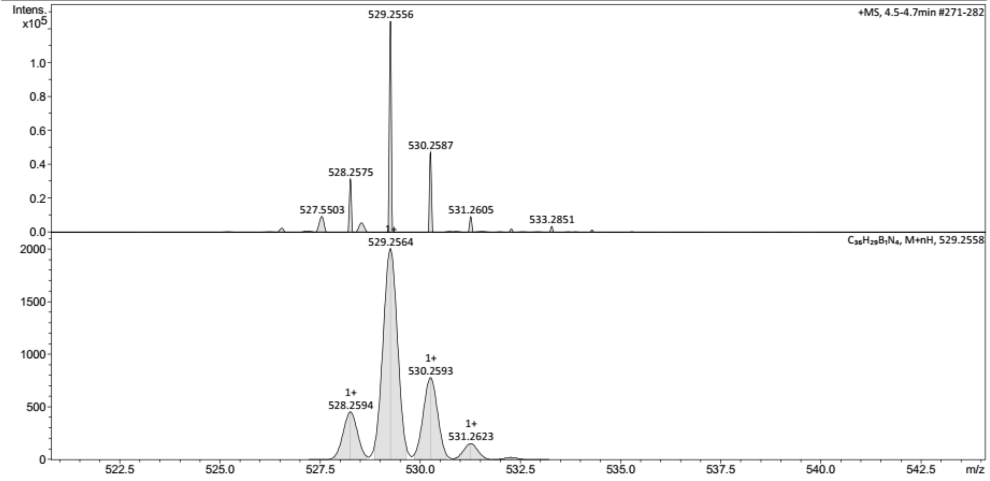

Bruker Compass DataAnalysis 4.1 printed: 2/2/2022 11:03:00 AM by: Bruker UK 1 of 1

Figure **S15**. HRMS of **AZB-TRZ**

Analysis type:

Single ☐ **Duplicate** ☑ **Triplicate** ☐

Analysis Result:

Element	Expected %	Found (1)	Found (2)	Found (3)
Carbon	81.82	81.75	82.07	
Hydrogen	5.53	5.70	5.76	
Nitrogen	10.60	10.40	10.43	
Oxygen				

Authorising Signature:

Date completed	21.03.22
Signature	[illegible]
comments	

Figure **S16**. Elemental analysis of **AZB-TRZ**

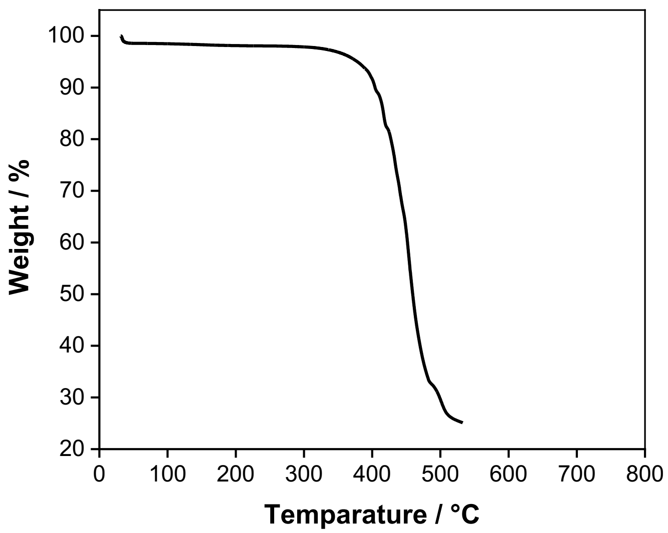

Figure **S17**. TGA Curve of **AZB-TRZ**

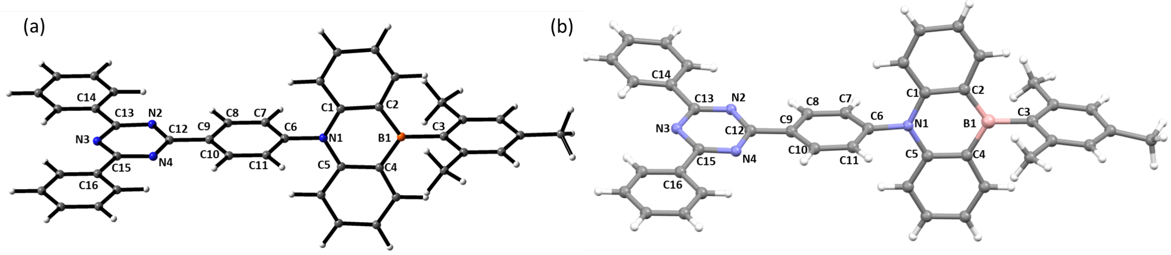

Figure **S18**. Selected atom numbering of (a) DFT-optimized structure (b) crystal structure.

Table **S1**. Selected bond lengths (Å), bond angles (°) and dihedral angles (°) of optimized structure and crystal structure.

Geometric parameters	Optimized structure	Crystal structure
C1-N1	1.392	1.406(2)
C5-N1	1.392	1.401(2)
C1-C2	1.416	1.416(2)
C5-C4	1.416	1.416(2)
C2-B1	1.528	1.530(2)
C4-B1	1.528	1.527(2)
B1-C3	1.572	1.578(2)
C6-N1	1.430	1.442(2)
C12-C9	1.478	1.481(2)
C12-N2	1.335	1.341(2)
C12-N4	1.335	1.347(2)
C13-N3	1.336	1.345(2)
C15-N3	1.336	1.339(2)
C13-C14	1.475	1.483(2)

C15-C16	1.475	1.489(2)
∠C1-N1-C5	122.9	123.3(1)
∠C1-N1-C6	118.0	118.5(1)
∠C5-N1-C6	118.0	118.2(1)
∠C2-B1-C3	122.4	120.8(1)
∠C3-B1-C4	122.4	124.4(1)
∠C2-B1-C4	115.3	114.8(1)
	Dihedral angles	
C1-N1-C6-C7	90.0	81.5(2)
C5-N1-C6-C11	90.0	81.3(2)
C8-C9-C12-N2	0.0	8.46(2)
C10-C9-C12-N4	0.0	7.43(2)

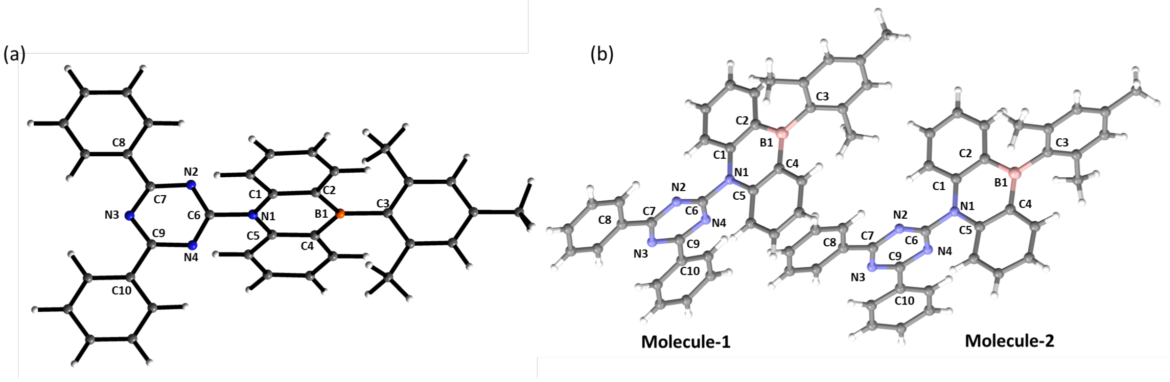

Figure **S19**. Selected atom numbering of (a) optimized structure (b) crystal structure (asymmetric unit contains two molecules).

Table **S2**. Selected bond lengths (Å), bond angles (°) and dihedral angles (°) of optimized structure and crystal structure.

Geometric parameters	Optimized structure	Crystal structure (Asymmetric unit has two molecules) Molecule-1	Molecule-2
C1-N1	1.389	1.393(3)	1.392(3)
C5-N1	1.389	1.388(2)	1.393(2)
C1-C2	1.414	1.411(3)	1.409(2)
C5-C4	1.414	1.412(2)	1.408(2)
C2-B1	1.529	1.528(2)	1.524(2)
C4-B1	1.529	1.531(3)	1.530(3)
B1-C3	1.572	1.582(3)	1.583(3)
C6-N1	1.424	1.435(2)	1.431(2)
C6-N2	1.323	1.317(2)	1.322(2)
C7-N3	1.335	1.335(3)	1.340(3)
C9-N3	1.335	1.333(2)	1.336(2)
C9-N4	1.344	1.345(2)	1.342(2)
C6-N4	1.323	1.327(2)	1.328(2)
C7-C8	1.471	1.470(2)	1.473(2)

C9-C10	1.471	1.484(3)	1.483(3)
∠C1-N1-C5	124.7	124.4(2)	124.1(2)
∠C1-N1-C6	117.6	117.3(2)	117.7(2)
∠C5-N1-C6	117.6	118.2(2)	118.1(2)
∠C2-B1-C3	112.3	122.0(2)	121.9(2)
∠C3-B1-C4	122.3	123.1(2)	122.8(2)
∠C2-B1-C4	115.4	114.9(2)	115.2(2)
Dihedral angles			
C1-N1-C6-N2	90.0	88.4(2)	95.9(2)
C5-N1-C6-N4	90.0	87.1(2)	99.2(2)

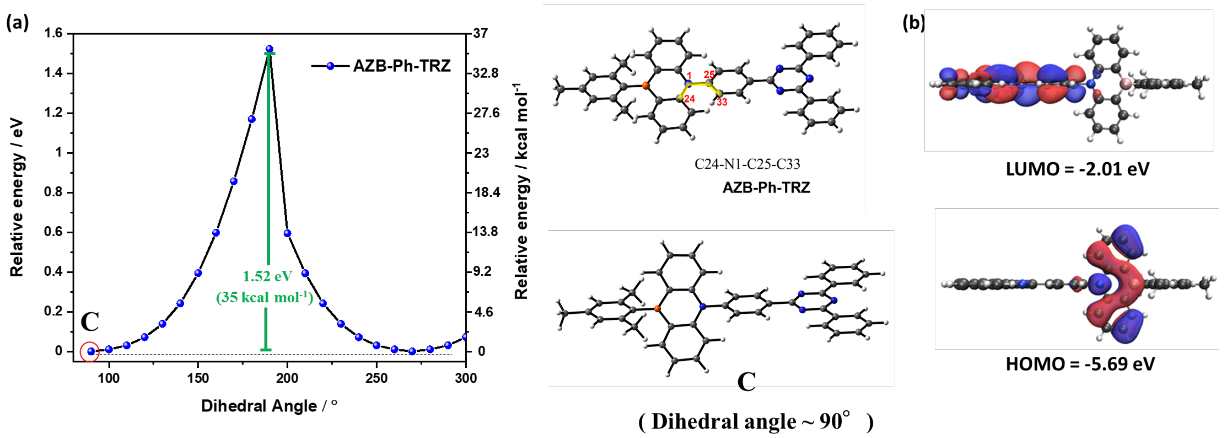

Figure **S20**. (a) Potential energy surface analysis, (b) FMOs of **AZB-Ph-TRZ**.

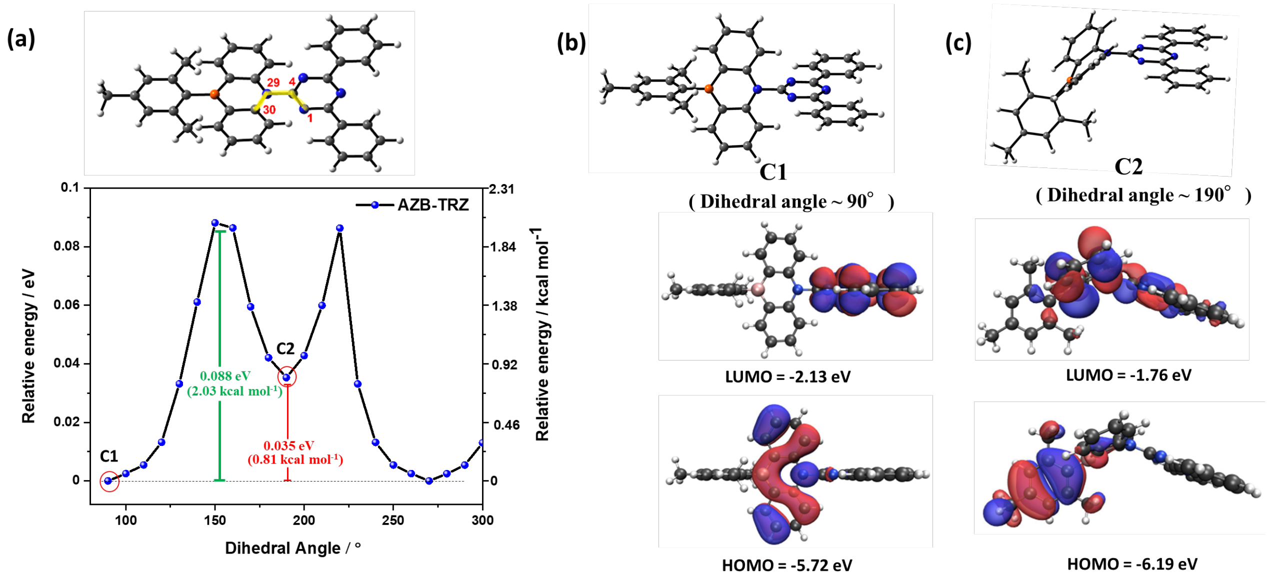

Figure **S21**. (a) Potential energy surface analysis and, FMOs of (b) orthogonal conformer (**C1**), and (c) bent conformer (**C2**) of **AZB-TRZ**.

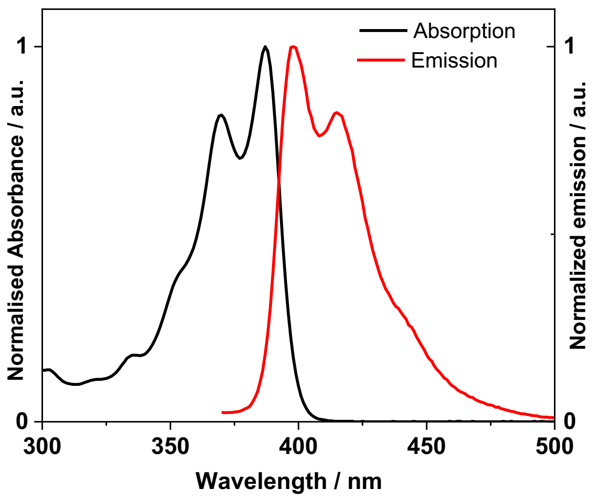

Figure **S22**. Absorption and fluorescence emission of azaborine donor (compound **2**) in toluene (λ_{exc} = 340 nm)

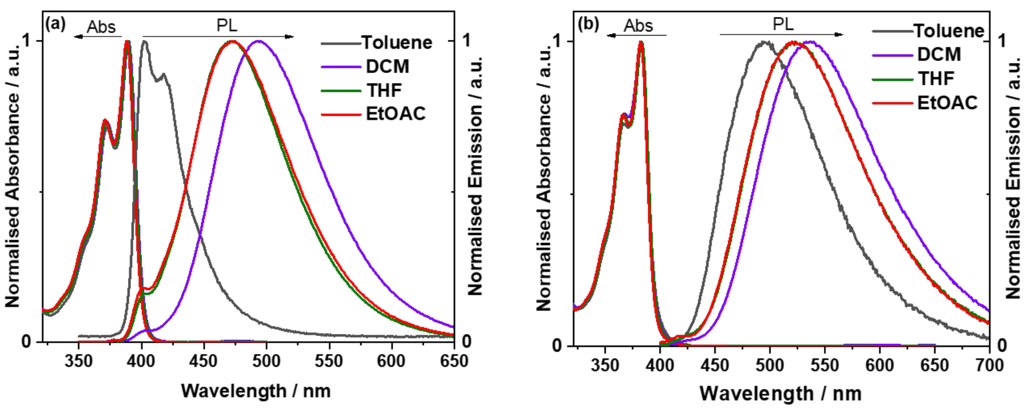

Figure **S23**. Absorption spectra and emission spectra of (a) **AZB-Ph-TRZ** and (b) **AZB-TRZ** measured in different solvents (λ_{exc} = 340 nm).

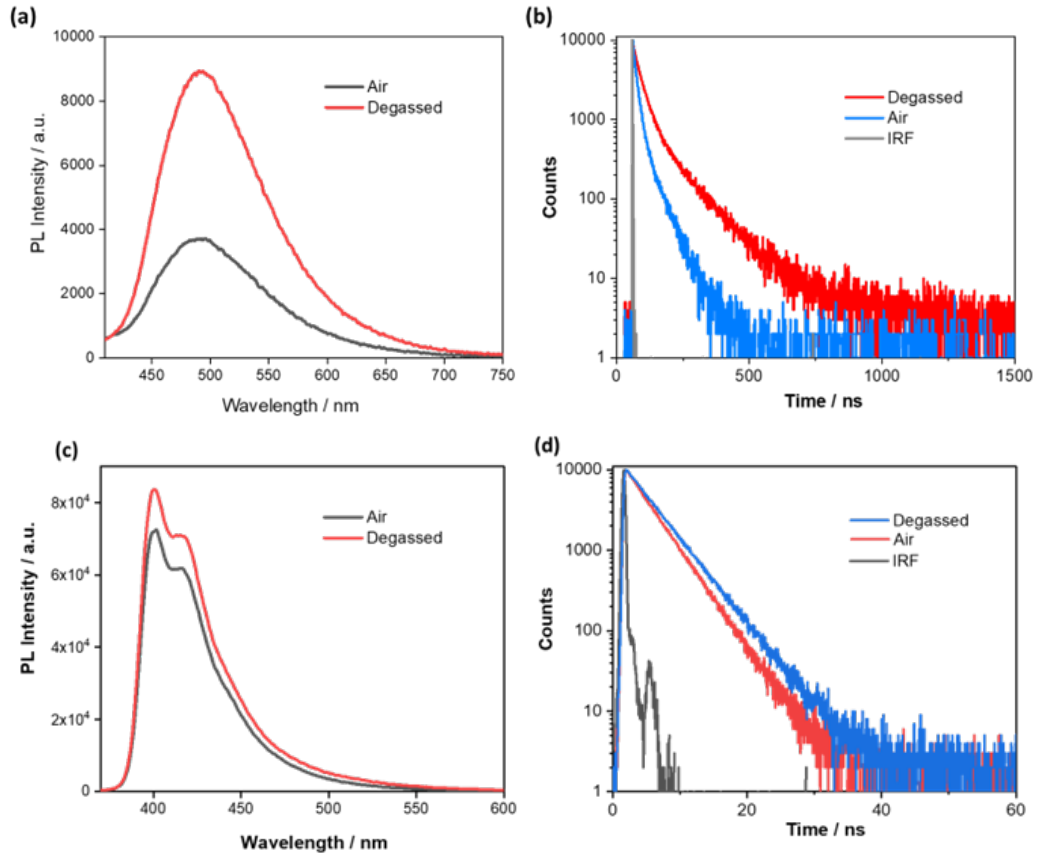

Figure **S24**. PL spectra and emission decays of (a,b) **AZB-TRZ** and (c,d) **AZB-Ph-TRZ** in toluene under degassed conditions and air.

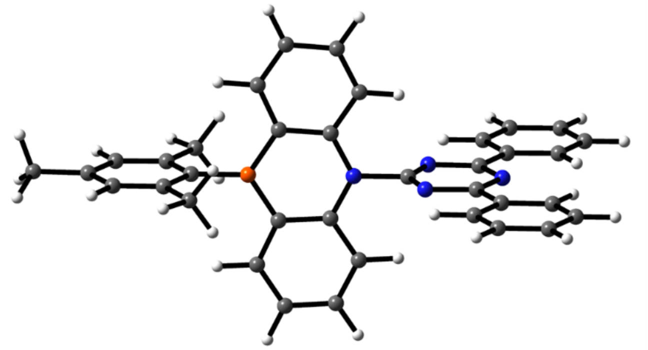

Orthogonal conformer

$S_1 = 2.16$ eV; $f = 0.0012$ ($S_1 \rightarrow S_0$)

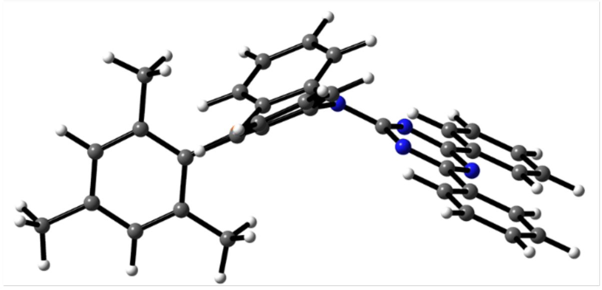

Bent conformer

$S_1 = 2.94$ eV; $f = 0.0001$ ($S_1 \rightarrow S_0$)

Figure **S25**. Optimized S_1 excited-state geometries of **AZB-TRZ** (conformer C1 and conformer C2 on the ground-state PES were taken as the input geometries), and their associated energies and oscillator strengths (*f*).

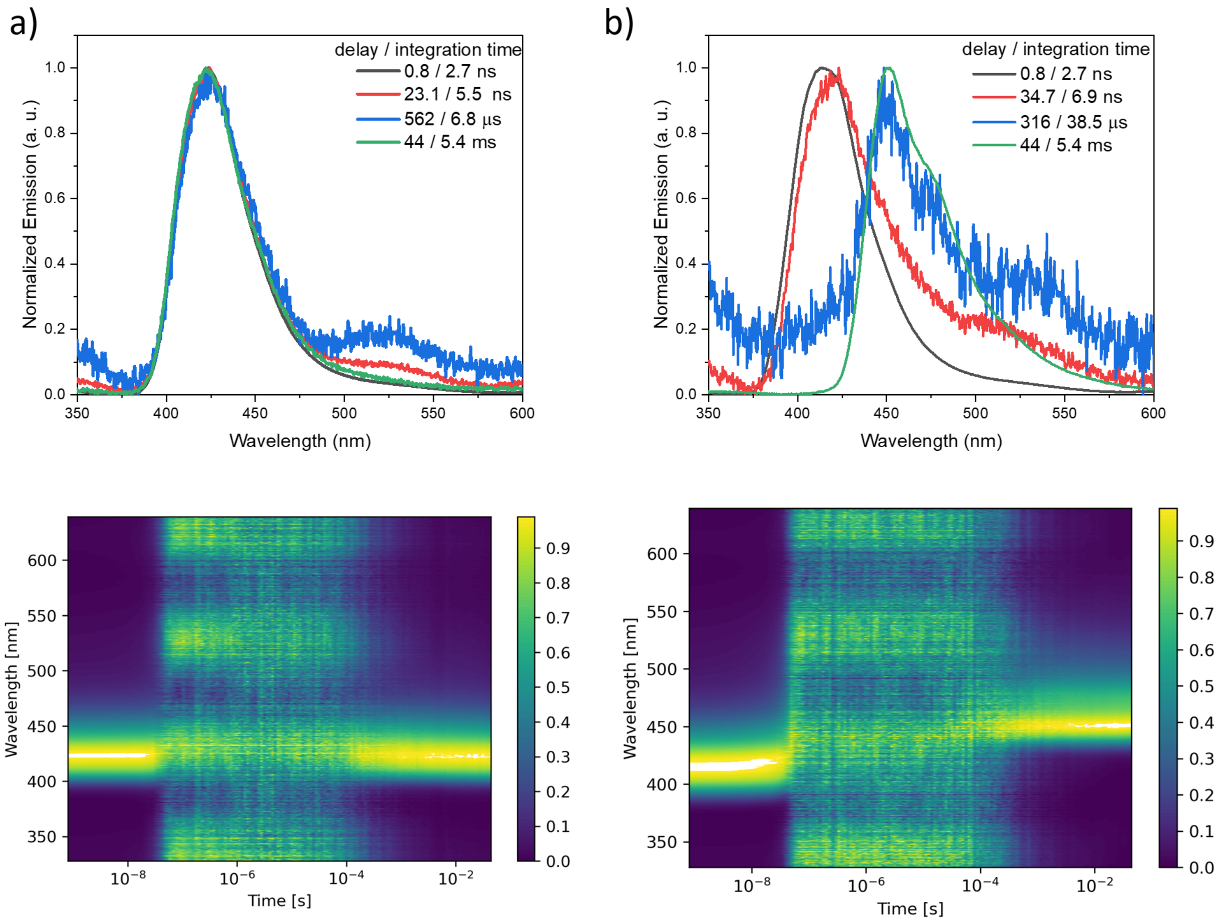

Figure **S26**. Time resolved spectra obtained at different delay/integration times for **AZB-Ph-TRZ** doped in zeonex (1 wt%) at (a) RT and (b) 80 K. Bottom panels represent the contour plots of time-resolved emission spectra at respective temperatures (regions between 100 ns and 100 µs at both RT and 80K represent hardware background collection, i.e., no detectable emission). λ_{exc} = 355 nm.

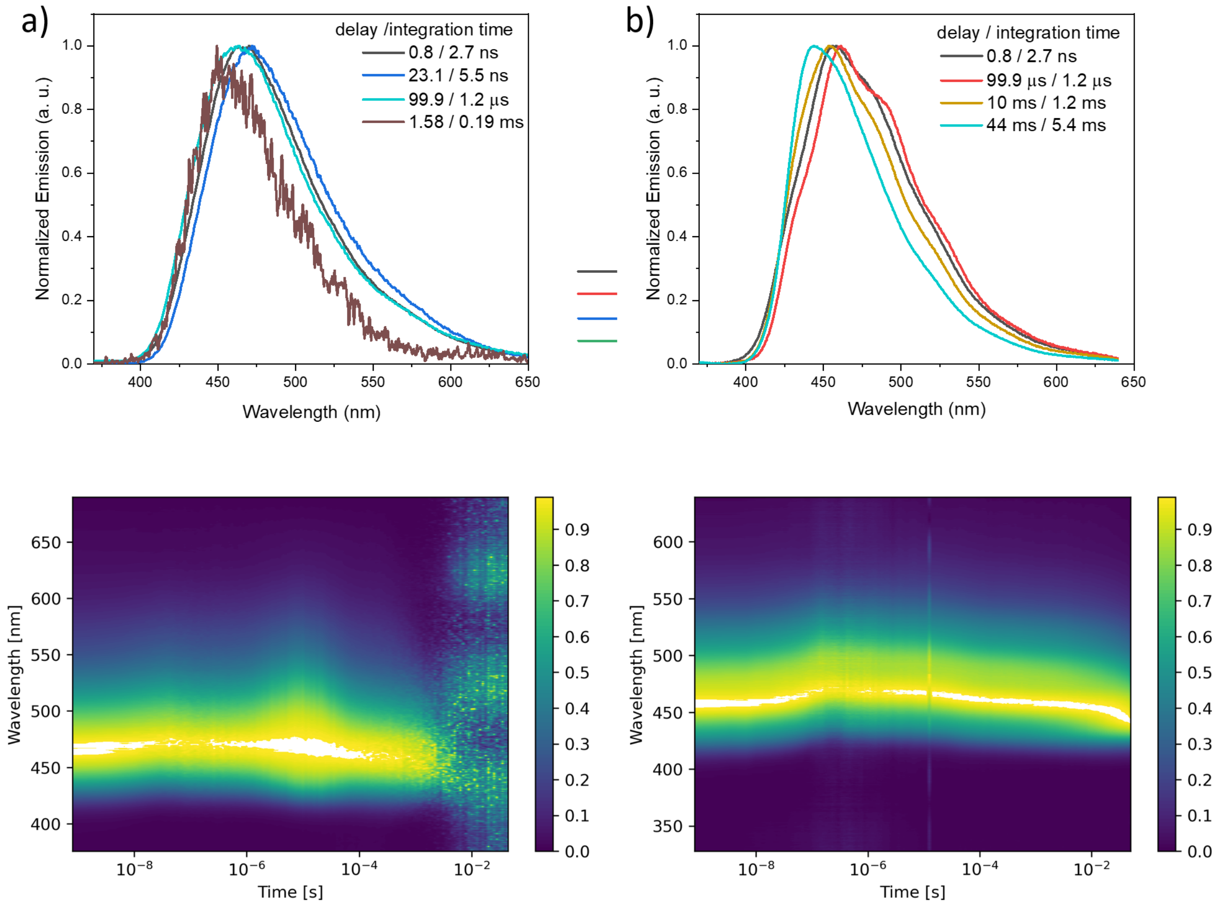

Figure **S27**. Time-resolved spectra obtained at different delay/integration times for **AZB-TRZ** doped in zeonex (1 wt%) at (a) RT and (b) 80 K. Bottom panels represent the contour plots of time-resolved emission spectra at respective temperatures. λ_{exc} = 355 nm.

Table **S3**. Fitted exponential lifetimes and rate constants of emission decays

**AZB-Ph-TRZ:**	τ_{PF} [a] / ns	τ_{DF} [a] / µs	k_F [b] / × 10^6 s^{-1}	k_{ISC} [b] / × 10^7 s^{-1}	k_{RISC} [b] / × 10^5 s^{-1}	Φ_{PL} (N_2/air) / %	S_1 / eV	T_1 / eV	ΔE_{ST} / eV
Zeonex	4.1	-*	-	-	-	-	3.16	2.91	0.25
DPEPO	4.3	14.3	-	-	-	20/17	3.04	2.89	0.15
mCP	6.5	-*	-	-	-	27/21	3.25	2.86	0.39
**AZB-TRZ:**	τ_{PF} [a] / ns	τ_{DF} [a] / µs	k_F [b] / × 10^6 s^{-1}	k_{ISC} [b] / × 10^7 s^{-1}	k_{RISC} [b] / × 10^5 s^{-1}	Φ_{PL} (N_2/air) / %	S_1 / eV	T_1 / eV	ΔE_{ST} / eV
Zeonex	15	12	3.0 ± 0.2	7.7 ± 0.3	57 ± 2	-	3.00	2.99	0.01
DPEPO	14	5.8	12 ± 0.5	5.5 ± 0.3	22 ± 12	41/36	2.94	2.96	0.02
mCP	26	1.1	8.6 ± 0.4	4.7 ± 0.1	49 ± 3	34/19	2.94	2.93	0.01

[a]amplitude-weighted average of lifetimes from bi-exponential fitting of PF or DF time regime; [b]rates from simultaneous kinetic fitting of PF and DF. Model assumptions in kinetic fitting make it only appropriate for TADF materials with strong DF and approximately single-exponential decay, therefore only applied to **AZB-TRZ** in this case. *strongly non-exponential

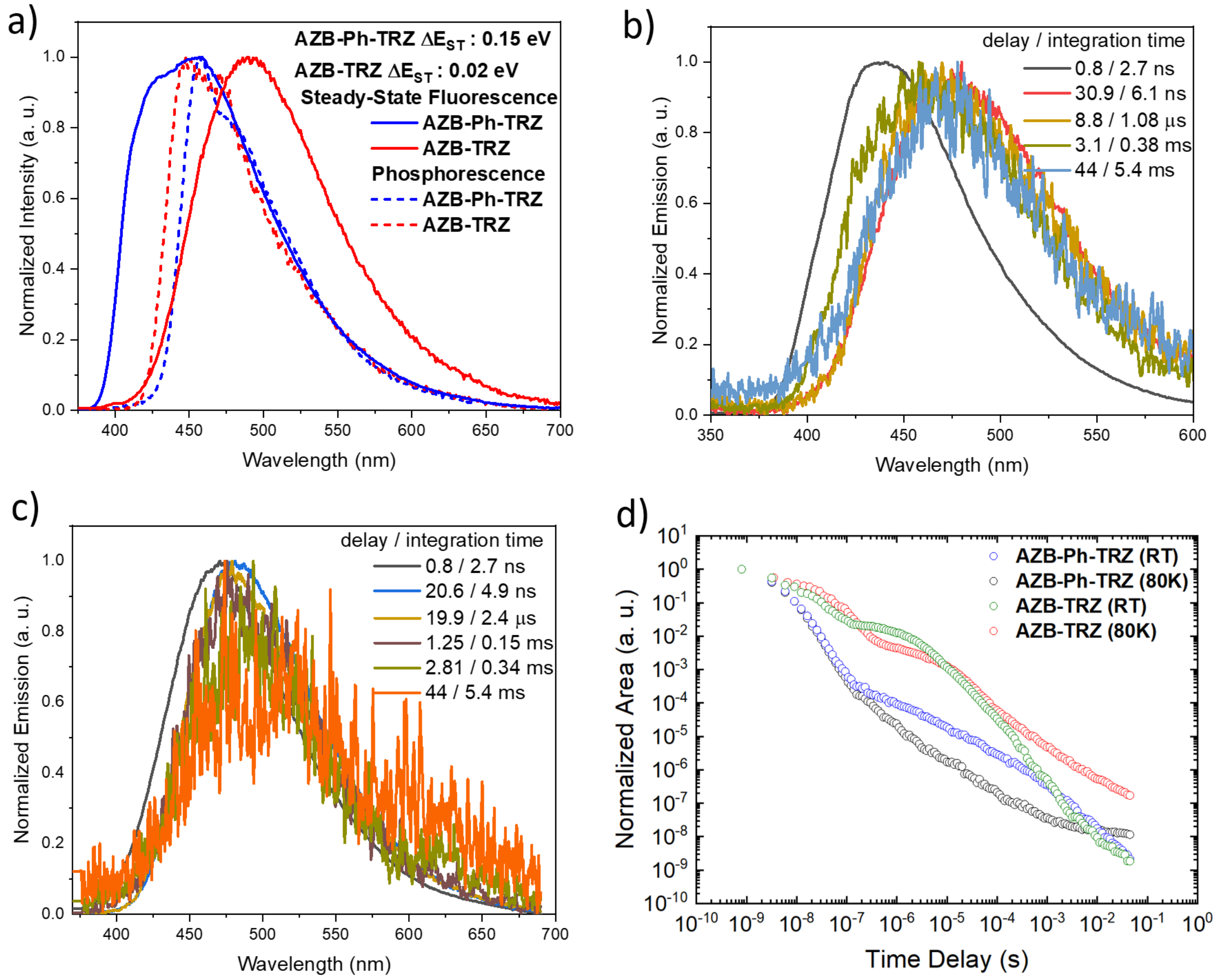

Figure **S28**. a) Steady-state fluorescence (ambient) and phosphorescence (delayed, delay time 80 ms and gate width 15 ms at 80 K) spectra of **AZB-Ph-TRZ** and **AZB-TRZ** films doped in DPEPO (10 wt%). b) Emission decay and time resolved spectra of the same films obtained at different delay times for **AZB-Ph-TRZ** and (c) **AZB-TRZ** RT. d) Time-resolved emission decay of **AZB-Ph-TRZ** and **AZB-TRZ** films doped in DPEPO (10 wt%) at room temperature and 80 K. λ_{exc} = 355 nm.

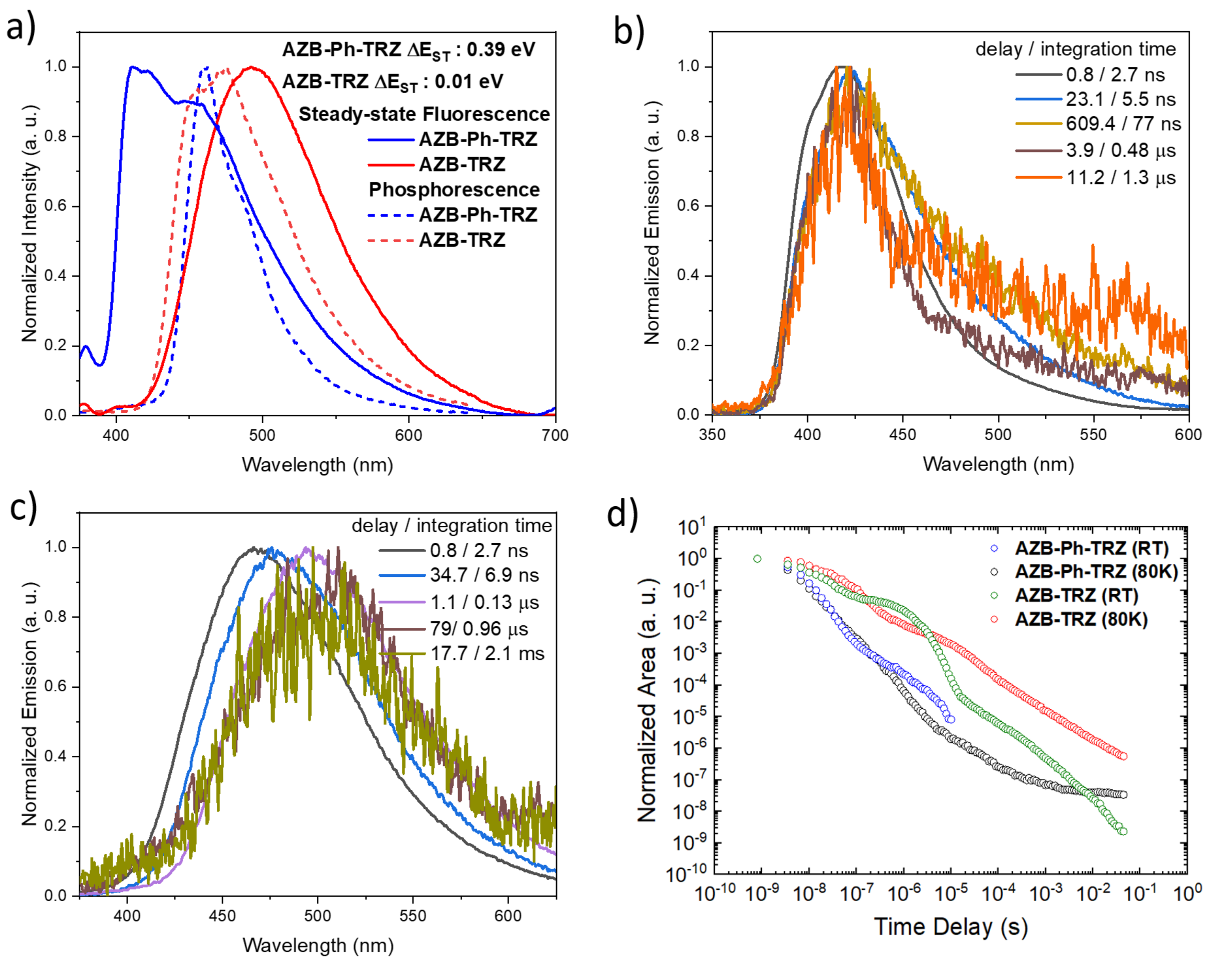

Figure **S29**. a) Steady-state fluorescence (ambient) and phosphorescence (delayed, delay time 80 ms and gate width 15 ms at 80 K) spectra of **AZB-Ph-TRZ** and **AZB-TRZ** films doped in mCP (10 wt%). b) Emission decay and time resolved spectra of the same films obtained at different delay times for **AZB-Ph-TRZ** and (c) **AZB-TRZ** RT. d) Time-resolved emission decay of **AZB-Ph-TRZ** and **AZB-TRZ** films doped in mCP (10 wt%) at room temperature and 80 K. λ_{exc} = 355 nm.

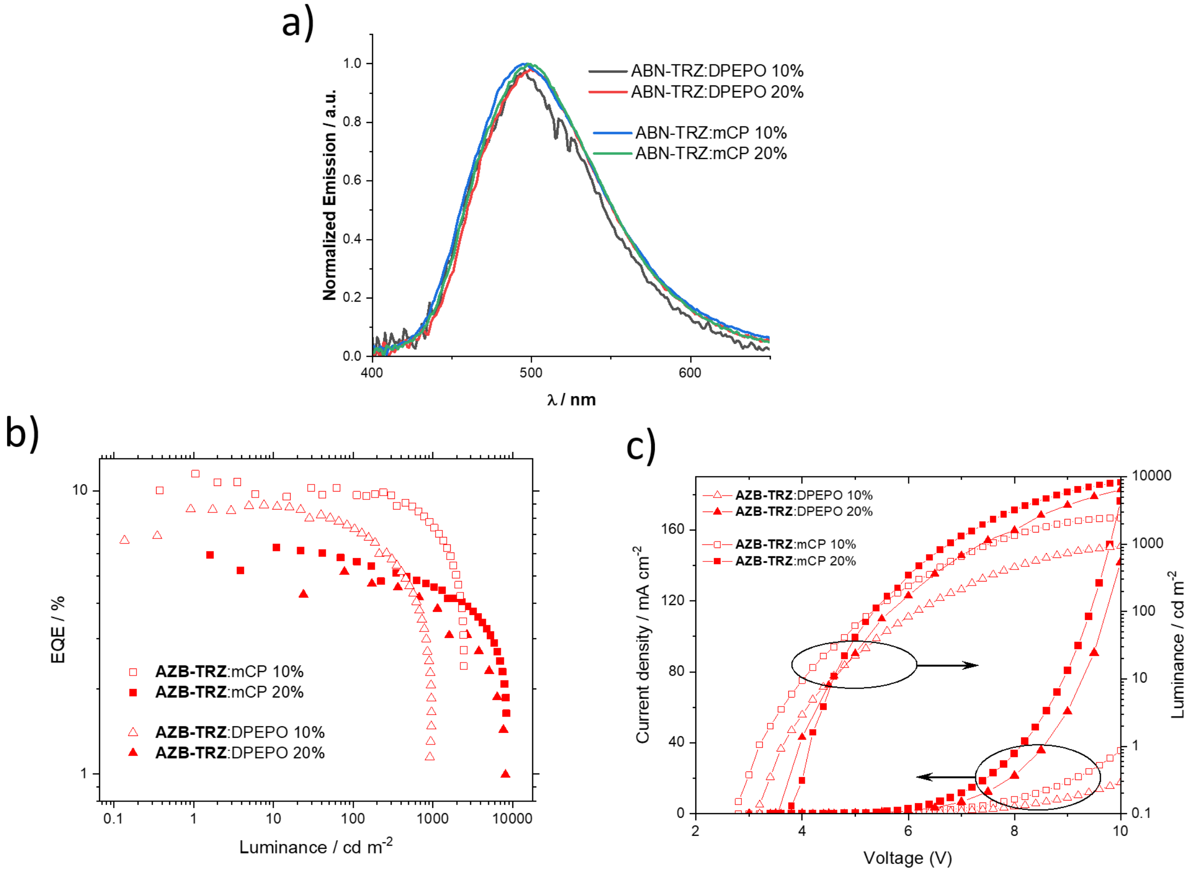

Figure **S30**. OLED performance comparisons for **AZB-TRZ** at different doping concentrations. a) EL spectra; b) Current and luminance at different voltages; c) External quantum efficiency at different luminances.
